# NEDD4L mediates ITGB4 ubiquitination and degradation to suppress esophageal carcinoma progression

**DOI:** 10.1186/s12964-024-01685-9

**Published:** 2024-06-03

**Authors:** Yijun Shi, Na Fang, Yutong Wu, Huiwen Xu, Anhui Ning, Wendi Zhang, Yiran Liu, Xiaobo Tao, Qiong Chen, Tian Tian, Lei Zhang, Minjie Chu, Jiahua Cui

**Affiliations:** 1 Department of Thoracic and Cardiovascular Surgery, The Affiliated People’s Hospital of Jiangsu University, Zhenjiang, 212002 China; 2 Department of Oncology, The Affiliated People’s Hospital of Jiangsu University, Zhenjiang, 212002 China; 3 Department of Epidemiology, School of Public Health, Nantong University, 9 Seyuan Road, Nantong, 226019 China; 4 Department of Oncology, The First Affiliated Hospital of Nanjing Medical University, Nanjing, 210029 China

**Keywords:** Esophageal carcinoma, NEDD4L, ITGB4, Ubiquitination

## Abstract

The ubiquitination-mediated protein degradation exerts a vital role in the progression of multiple tumors. NEDD4L, which belongs to the E3 ubiquitin ligase NEDD4 family, is related to tumor genesis, metastasis and drug resistance. However, the anti-tumor role of NEDD4L in esophageal carcinoma, and the potential specific recognition substrate remain unclear. Based on public esophageal carcinoma database and clinical sample data, it was discovered in this study that the expression of NEDD4L in esophageal carcinoma was apparently lower than that in atypical hyperplastic esophageal tissue and esophageal squamous epithelium. Besides, patients with high expression of NEDD4L in esophageal carcinoma tissue had longer progression-free survival than those with low expression. Experiments in vivo and in vitro also verified that NEDD4L suppressed the growth and metastasis of esophageal carcinoma. Based on co-immunoprecipitation and proteome analysis, the NEDD4L ubiquitination-degraded protein ITGB4 was obtained. In terms of the mechanism, the HECT domain of NEDD4L specifically bound to the Galx-β domain of ITGB4, which modified the K915 site of ITGB4 in an ubiquitination manner, and promoted the ubiquitination degradation of ITGB4, thus suppressing the malignant phenotype of esophageal carcinoma.

**Supplementary Information:**

The online version contains supplementary material available at 10.1186/s12964-024-01685-9.

## Introduction

Esophageal carcinoma (ESCA) is one of the commonly seen malignant tumors globally, its morbidity ranks the 7th place among malignant tumors worldwide, and it takes the 6th place among causes of cancer-related mortality [1]. The morbidity of esophageal carcinoma exhibits obvious regional divergence, notably, the esophageal carcinoma cases in Asia occupy about 78% of the global esophageal carcinoma cases, among them, 49% of the total Asian esophageal carcinoma cases come from China. Esophageal carcinoma has become one of the non-negligible diseases threatening the health of Chinese population [2]. Esophageal carcinoma can be mainly classified as two major pathological types, namely, esophageal squamous cell carcinoma (ESCC) and esophageal adenocarcinoma (EAC), of which, ESCC accounts for as high as 70% of esophageal carcinoma cases [3]. It is difficult to discover esophageal carcinoma due to its lack of early typical symptom, the application of endoscopy is limited because it is likely to induce patient discomfort, and 60–70% of patients are already at the middle-to-advanced stages when they are first diagnosed [4]. At present, surgery combined with radiochemotherapy has been a standard treatment mode for locally-advanced esophageal carcinoma, while the application of targeted therapy and immune checkpoint inhibitors in esophageal carcinoma patients is relatively limited [5, 6]. On the whole, patients with esophageal carcinoma in China have poor prognosis, with a 5-year survival rate of only 20.9% [7]. Consequently, investigating the molecular mechanism affecting the progression of esophageal carcinoma at the molecular level, and searching for the biomarkers and specific therapeutic targets for predicting the efficacy of esophageal carcinoma treatment is of great significance for improving the prognosis of esophageal carcinoma patients.

Ubiquitin (UB) is a type of highly conserved small molecular protein, with the molecular mass of 8.5 ku. It consists of 76 amino acid residues and is extensively distributed in eukaryotic cells [8]. Ubiquitination allows for the selective degradation of a majority of proteins in the eukaryotic cells [9]. The protein ubiquitination modification process exerts an extremely important effect on regulating various cellular biological functions, including cell cycle, DNA damage repair, signal transduction and membrane localization of various proteins [10]. It is recently discovered that protein ubiquitination exerts a vital role in tumor proliferation, invasion and metastasis [11–15].

In the ubiquitination-modified protein degradation process, E3 ubiquitin ligase plays a role of efficiently regulating and specifically recognizing substrate in the cascade process [16]. The change of E3 activity, and the resultant changes of the ubiquitin-proteasome system, protein stabilization regulation, protein transport and other ubiquitin-driven pathways, have affected numerous basic biological processes, including tumor progression [17–19]. E3 ubiquitin ligases can be broadly classified as four types, including HECT type, U-box type, RING finger type and RBR type. Among them, HECT is the greatest E3 ubiquitin ligase family [20]. NEDD4L (neural precursor cell expressed developmentally down-regulated 4-like) is an important member of the NEDD4 family, which possesses the HECT, WW and C2 domains, directly binds to the substrate and determines the specificity of the ubiquitin system [21]. The NEDD4L-involved ubiquitination modification exerts a crucial effect on multiple tumors. Some research has suggested that, NEDD4L promotes the ubiquitination degradation of HIF-1α to suppress the malignant phenotype of colorectal carcinoma [22]. NEDD4L contributes to the ubiquitination degradation of GPX4, induces the ferroptosis in non-small cell lung cancer, and thereby suppresses tumor progression [23]. However, the anti-tumor role of NEDD4L in esophageal carcinoma and the possible specifically recognized substrate remain unclear.

Based on GEO database data, it was discovered in this study that, the expression of NEDD4L in esophageal carcinoma was apparently lower than that in atypical hyperplastic esophageal tissue and esophageal squamous epithelium. Additionally, patients with high expression of NEDD4L in esophageal carcinoma tissue had longer progression-free survival than those with low expression. In experiments in vivo and in vitro, NEDD4L suppressed the growth and metastasis of esophageal carcinoma. Mechanically, the HECT domain of NEDD4L specifically bound to the Galx-β domain of ITGB4, which modified the K915 site of ITGB4 in an ubiquitination manner, and promoted the ubiquitination degradation of ITGB4, thus suppressing the malignant phenotype of esophageal carcinoma. This study suggests that NEDD4L may be the early diagnostic molecular biomarker and potential therapeutic target for esophageal carcinoma.

## Results

### NEDD4L was positively correlated with the improved prognosis of esophageal carcinoma

**Fig. 1:**
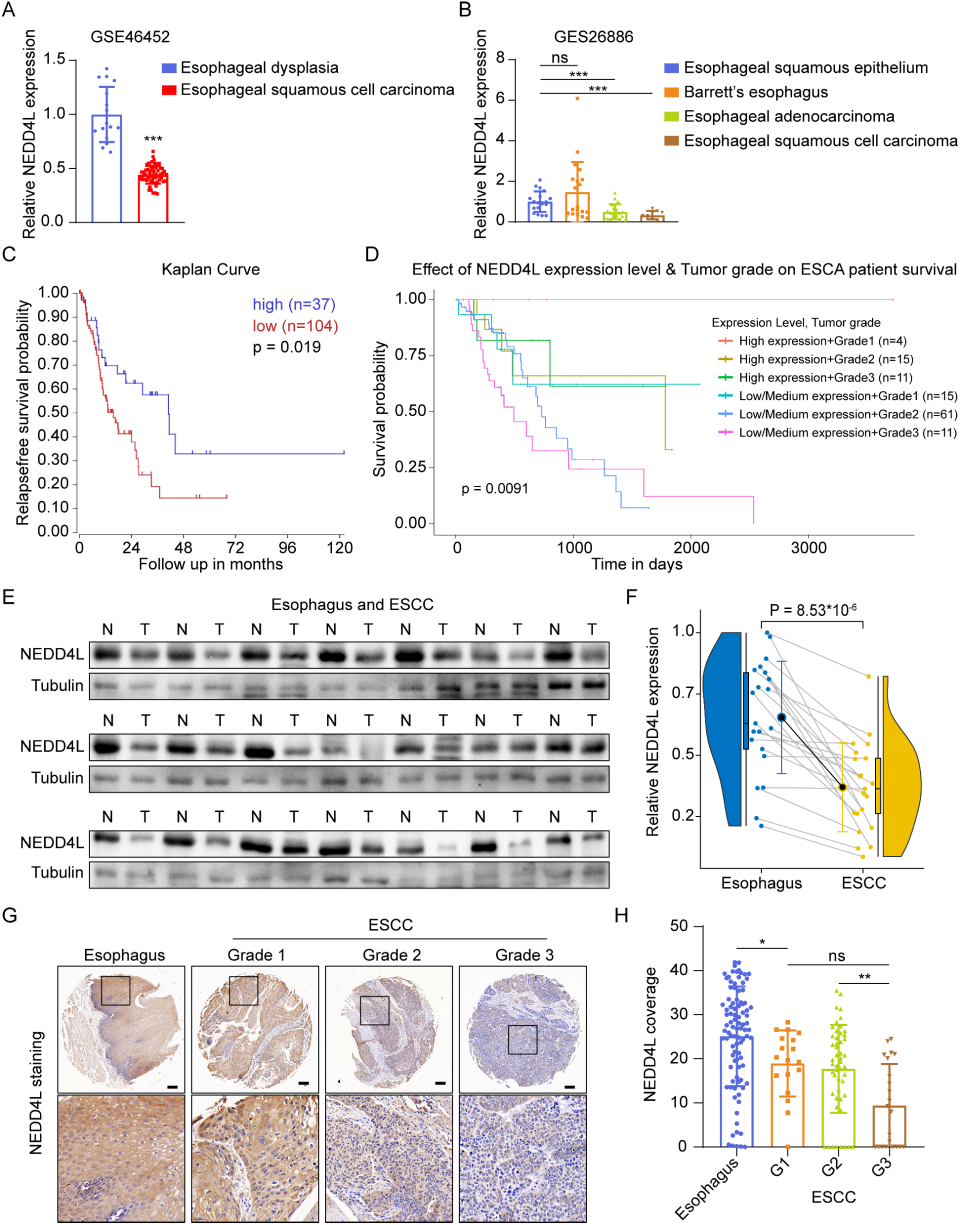
NEDD4L was positively associated with improved prognosis of esophageal carcinoma patients. **(A-B)** The mRNA expression of NEDD4L was interrogated in unpaired cohorts GSE46452 database (59 Esophageal Squamous Cell Carcinoma samples, 16 non-cancerous Esophageal Dysplasia samples) **(A)** and GSE26886 database (20 Barrett’s esophagus samples, 21 esophageal adenocarcinoma samples, 19 esophageal squamous epithelium samples, and 9 esophageal squamous cell carcinoma samples) **(B)**. **(C-D) (C)** Kaplan–Meier curves depicted progression-free survival according to the NEDD4L expression of ESCA cohort. **(D)** Kaplan–Meier curves depicted Overall Survival in indicated groups. **(E-F)** The proteins extracted from ESCC tissues and paired esophagus tissues were assessed by Western blot analysis **(E)**; quantitation of NEDD4L protein concentrations is shown **(F)**. (*n* = 21). **(G-H)** Representative immunohistochemical staining of NEDD4L in different grades of esophageal carcinoma. The dashed box is further magnified by 4.5× **(G)**. Quantification of NEDD4L intensity **(H)**. Scale bars: 100 μm. (*P value < 0.05; **P < 0.01; ***P < 0.001; ns, no significance)

To evaluate the role of NEDD4L expression on the prognosis of esophageal carcinoma patients, we downloaded esophageal carcinoma and para-carcinoma expression data from GEO database to explore the expression of NEDD4L in cancer and para-carcinoma tissue samples. The results suggested that, the expression of NEDD4L in normal esophagus was apparently higher than that in esophageal carcinoma tissue (GSE46452, Fig. 1A). GES26886 data further indicated that the expression of NEDD4L in normal esophagus and Barrett’s esophageal hyperplastic tissue apparently increased relative to that in EAC and ESCC (Fig. 1B). Kaplan-Meier method was used for survival analysis of 141 esophageal carcinoma patients, the results suggested that patients with high NEDD4L expression had significantly longer progression-free survival than those with low expression (Fig. 1C). Moreover, with the deterioration of malignancy grade of esophageal carcinoma, the patient survival markedly decreased, while high NEDD4L expression extended the patient survival (Fig. 1D). Subsequently, we collected 21 pairs of surgical tissue samples from ESCC patients for Western blot assay, and obtained 96 pairs of surgical tissue samples from ESCC patients for immunohistochemistry. Results of Western blot assay suggested that the expression of NEDD4L in ESCC was apparently lower than that in para-carcinoma tissues (Fig. 1E, F). Immunohistochemistry results also came to consistent conclusions. Moreover, NEDD4L expression decreased with the increased in ESCC malignancy grade (Fig. 1G, H and Supplementary Table 1). The above results indicate that NEDD4L is positively correlated with the improved prognosis of esophageal carcinoma.

### NEDD4L suppressed the malignant phenotype of esophageal carcinoma in vivo and in vitro

**Fig. 2:**
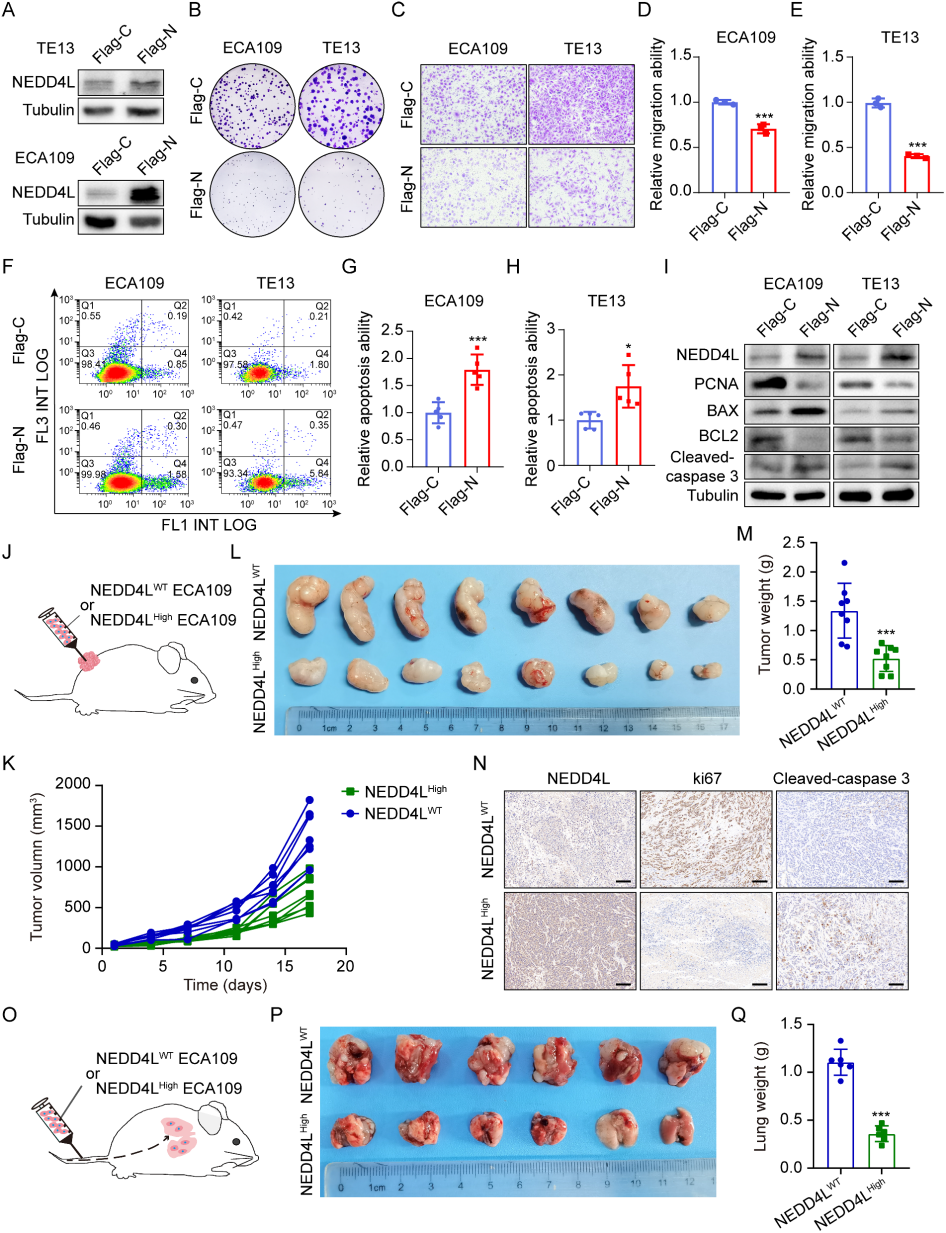
NEDD4L suppressed the malignant phenotype of esophageal carcinoma in vivo and in vitro. **(A)** Representative Western Blot analysis indicated the efficiency of NEDD4L overexpression after transfection with Flag-NEDD4L plasmid in TE13 and ECA109 cells. **(B)** The representative images of Colony formation after transfection with Flag-NEDD4L plasmid in TE13 and ECA109 cells. **(C-E)** The representative images and quantitative data of transwell assays after transfection with Flag-NEDD4L plasmid in TE13 and ECA109 cells. (*n* = 3). **(F-H)** Flow cytometric assay of TE13 and ECA109 cells after transfection with Flag-NEDD4L plasmid in TE13 and ECA109 cells. Data are mean ± standard deviation. (*n* = 5). **(I)** Protein expression of proliferation-related (PCNA) and apoptosis-related proteins (BAX, BCL2, Cleaved-Caspase 3) were detected by western blot analysis after transfection with Flag-NEDD4L plasmid in TE13 and ECA109 cells. **(J-N) (J)** Schematic representation of the NEDD4L^WT^ or NEDD4L^High^ ECA109 cells allograft-bearing model (*n* = 8). The tumor growth curves **(K)**, representative images of subcutaneous tumors **(L)**, and tumor weight **(M)** in indicated groups. **(N)** Representative immunohistochemical staining of NEDD4L, ki67 and Cleaved-Caspase 3 in the tumor nodules in indicated groups. scale bars: 100 μm. **(O-Q) (O)** Schematic representation of the NEDD4L^WT^ or NEDD4L^High^ ECA109 cells lung metastasis model (*n* = 6). The representative lung metastasis images **(P)** and lung weight **(Q)** in indicated groups. (*P value < 0.05; ***P < 0.001)

To verify the impact of NEDD4L on the malignant phenotype of esophageal carcinoma, we constructed the NEDD4L high-expression TE13 and ECA109 cell lines for cloning and Transwell assays (Fig. 2A). The results indicated that, TE13 and ECA109 cells with high NEDD4L expression had apparently decreased proliferation abilities relative to those with low NEDD4L expression (Fig. 2B). In migration assay, TE13 and ECA109 cells with high NEDD4L expression showed markedly reduced migration abilities compared with those with low NEDD4L expression (Fig. 2C-E). Flow cytometry further demonstrated that TE13 and ECA109 cells with high NEDD4L expression exhibited apparently increased apoptotic abilities compared with cells with low NEDD4L expression (Fig. 2F-H). The cell proliferation and apoptosis markers were detected through Western blot assay, as a result, TE13 and ECA109 cells with high NEDD4L expression showed down-regulation of PCNA and BCL2, while up-regulation of BAX and cleaved caspase3 (Fig. 2I).

To further explore the antitumor role of NEDD4L in vivo, we constructed the esophageal carcinoma xenograft model. First of all, NEDD4L^WT^/NEDD4L^High^ ECA109 cells at the logarithmic growth phase were injected into nude mice subcutaneously to construct the subcutaneous esophageal carcinoma tumor bearing model (Fig. 2J). The growth curve of xenograft esophageal carcinoma in mice is shown in Fig. 2K. Compared with NEDD4L^WT^ ECA109 cells-inoculated mice, the average tumor volume in mice inoculated with NEDD4L^High^ ECA109 cells increased at a slow rate. At the end of the mouse model, data verified that the tumor weight of mice inoculated with NEDD4L^High^ ECA109 cells apparently decreased, while NEDD4L inhibited 60.9% of esophageal carcinoma growth (Fig. 2L, M). As revealed by immunohistochemistry analysis of subcutaneous xenograft tissue in nude mice, the ki67 expression in nude mouse subcutaneous xenograft of NEDD4L^High^ group was lower than that in NEDD4L^WT^ group, while the cleaved caspase3 expression in subcutaneous xenograft of NEDD4L^High^ group was higher than that in NEDD4L^WT^ group (Fig. 2N). Afterwards, NEDD4L^WT^/NEDD4L^High^ ECA109 cells at the logarithmic growth phase were injected via the tail vein to construct the esophageal carcinoma metastasis model (Fig. 2O). On day 49 after cell inoculation, the model was terminated and lung metastasis in mice was evaluated. As shown in the Fig. 2P, Q, compared with mice inoculated with NEDD4L^WT^ ECA109 cells, the lung metastasis in those inoculated with NEDD4L^High^ ECA109 cells reduced by 67.5%. These results demonstrated that, NEDD4L suppresses the growth and metastasis of esophageal carcinoma in vivo and in vitro.

### NEDD4L promoted ITGB4 ubiquitination degradation to suppress the malignant phenotype of esophageal carcinoma

**Fig. 3:**
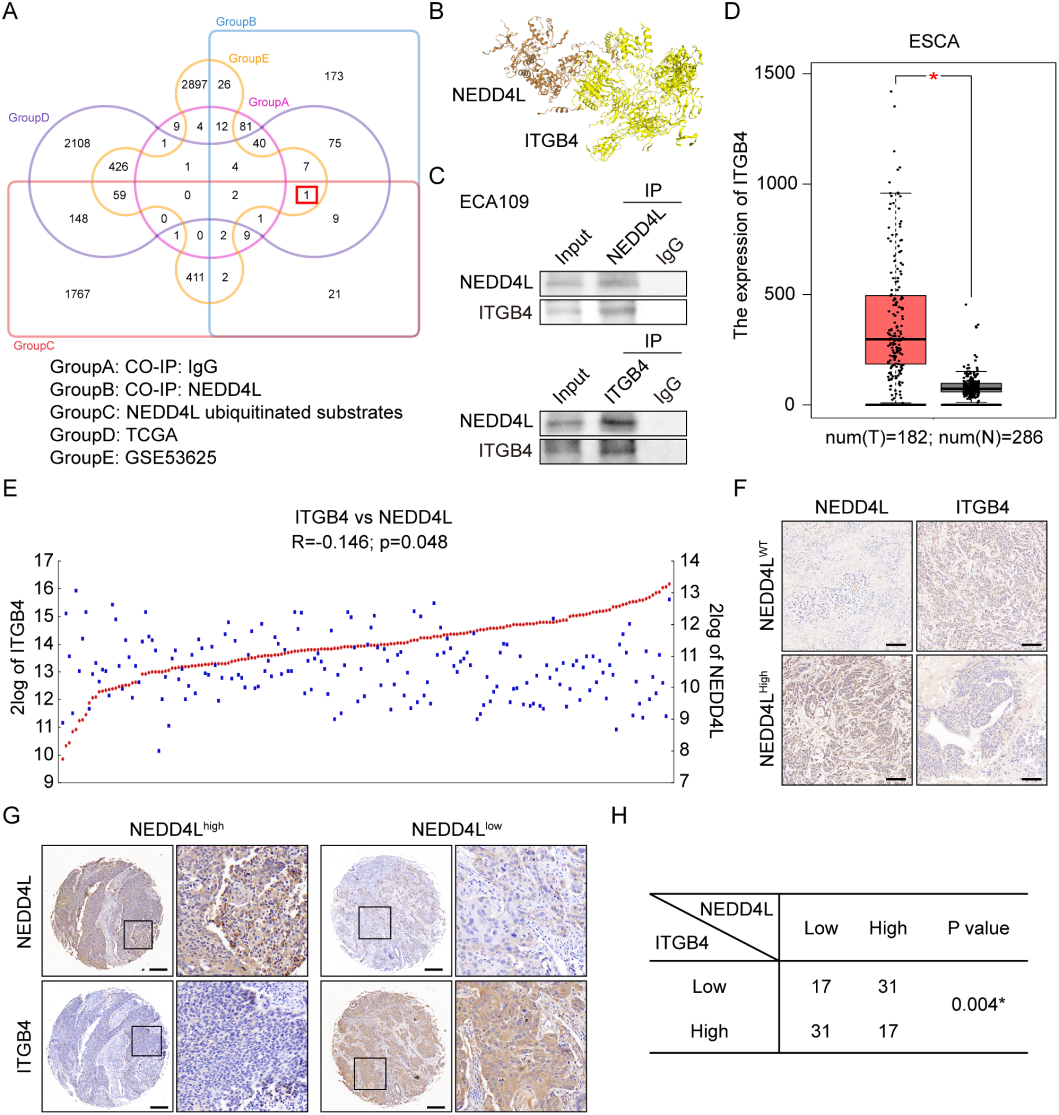
ITGB4 was negatively correlated with the expression of NEDD4L in ESCA. **(A)** Venn diagram for screening NEDD4L ubiquitination substrates in ESCA. **(B)** Molecular docking between NEDD4L and ITGB4 was determined via the HDCOK server. **(C)** Analysis of the interactions between NEDD4L and ITGB4 in ECA109 cell; by western blot analysis. **(D)** The expression of ITGB4 in ESCA was analyzed by GEPIA online database. **(E)** The correlation of NEDD4L and ITGB4 expression was determined by R2: Genomics Analysis and Visualization Platform. **(F)** Representative immunohistochemical staining of NEDD4L and ITGB4 in the ECA109 tumor nodules in indicated groups. scale bars: 100 μm. **(G-H) (G)** Representative immunohistochemical staining of NEDD4L and ITGB4 in esophageal carcinoma patients (The dashed box is further magnified by 4.7×). scale bars: 50 μm. **(H)** The expression levels of NEDD4L and ITGB4 were divided by the median immunohistochemical scores. 2 × 2 Table immunohistochemical results. The P-value of the correlation was calculated by Pearson x^2^ test. (*P value < 0.05)

To explore the role of NEDD4L-regulated ubiquitination modification in suppressing the malignant phenotype of esophageal carcinoma, we screened the NEDD4L ubiquitination-degraded protein. First, ECA109 cells were performed co-immunoprecipitation assay and protein mass spectrometry analysis to obtain 314 proteins that specifically bind to NEDD4L protein. Afterwards, the protein library (b = 2433 proteins) under the ubiquitination degradation by NEDD4L was downloaded from UbiBrowser database (http://ubibrowser.bio-it.cn/). The intersection was taken to obtain 33 NEDD4L-specific ubiquitination degraded proteins in esophageal carcinoma. Through the esophageal carcinoma TCGA database and GEO database (GSE53625), 492 esophageal carcinoma-related genes were screened. Finally, the protein ITGB4, which was related to esophageal carcinoma progression and could be degraded by NEDD4L through ubiquitination, was screened (Fig. 3A and Fig. S1). Then, NEDD4L-ITGB4 protein docking analysis was completed by HDOCK SERVER. The results indicated that there was a stable interaction pattern between NEDD4L and ITGB4 (Fig. 3B and Fig. S2). In addition, co-immunoprecipitation assay was conducted in ECA109 and TE13 cells, which also verified the interaction between NEDD4L and ITGB4 (Fig. 3C). Esophageal carcinoma TCGA data revealed that, the expression of ITGB4 in esophageal carcinoma tissue was apparently higher than that in normal esophageal tissue (Fig. 3D). At the same time, the expression of NEDD4L was negatively correlated with ITGB4 expression in esophageal carcinoma (Fig. 3E). According to immunohistochemistry analysis on 96 pairs of surgical tissues from esophageal carcinoma patients, the ITGB4 expression increased with the increase in esophageal carcinoma stage and grade (Supplementary Table 2). In the subcutaneous esophageal carcinoma tumor bearing model (Fig. 3F) and 96 pairs of surgical tissue samples from esophageal carcinoma patients (Fig. 3G, H), immunohistochemistry analysis further verified that NEDD4L was negatively correlated with ITGB4 expression. These data support the notion that NEDD4L may degrade ITGB4 through ubiquitination to suppress the malignant phenotype of esophageal carcinoma.

**Fig. 4:**
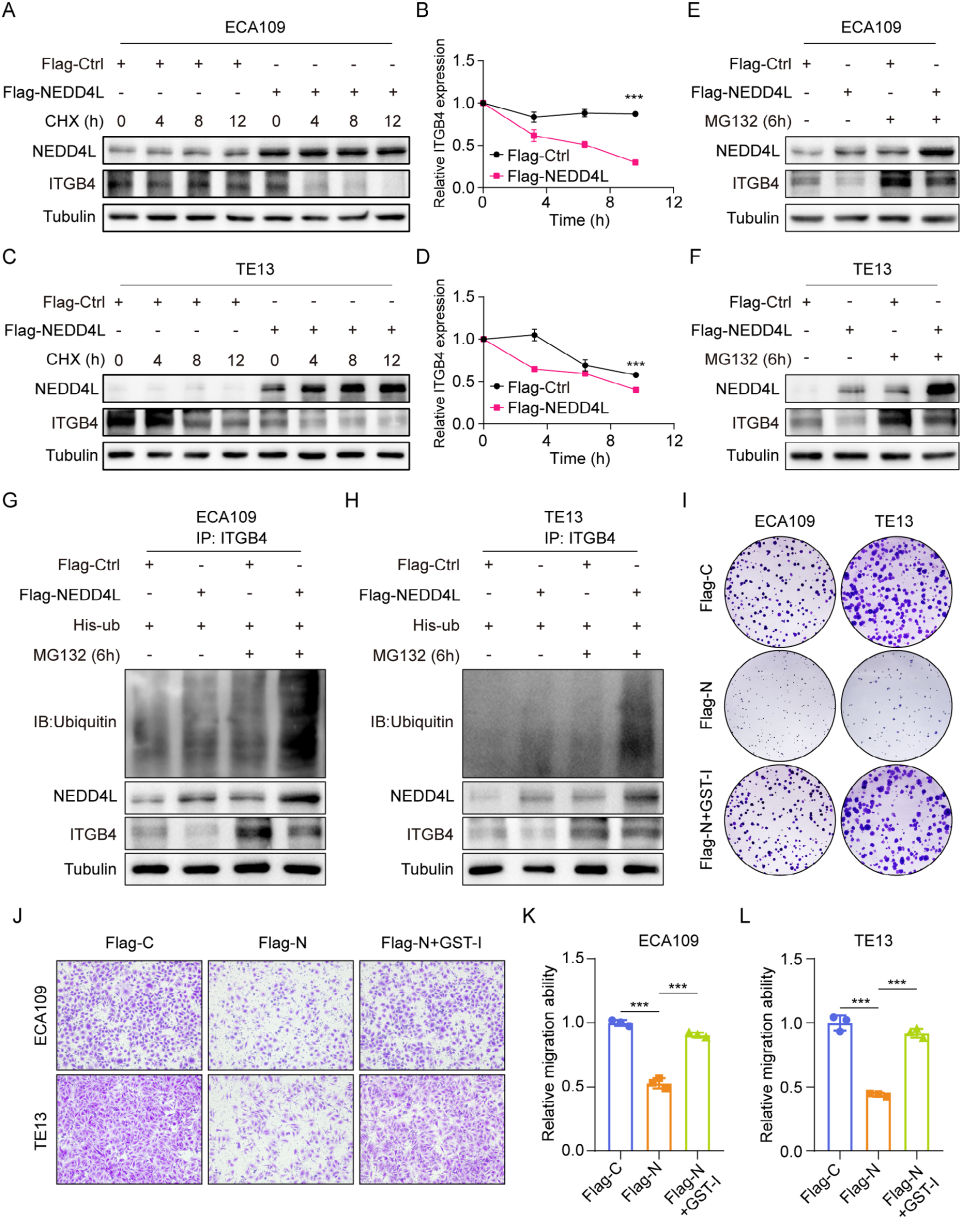
NEDD4L ubiquitinates ITGB4 in ESCA. **(A-D)** ITGB4 stability assay. The NEDD4L^WT^ or NEDD4L^High^ ECA109 **(A)** and TE13 **(C)** cells were treated with CHX for indicated time; expression of ITGB4 was determined by western blot analysis. The time-course intensities of the ITGB4 protein in ECA109 **(B)** and TE13 **(D)** cells (*n* = 3). **(E-F)** The NEDD4L^WT^ or NEDD4L^High^ ECA109 **(E)** and TE13 **(F)** cells were treated with MG132 for 6 h; ITGB4 was determined by western blot analysis. **(G-H)** The ECA109 **(G)** and TE13 **(H)** cells were treated with MG132 for 6 h; ubiquitinations and expressions of ITGB4 was determined by Co-IP and western blot analysis, respectively. **(I)** The representative images of Colony formation of TE13 and ECA109 cells in indicated groups. **(J-L)** The representative images and quantitative data of transwell assays in TE13 and ECA109 cells in indicated groups (*n* = 3). (***P value < 0.001)

To verify this hypothesis, we added cycloheximide (CHX, a protease synthesis inhibitor) in NEDD4L^WT^ and NEDD4L^High^ ECA109 and TE13 cells to treat for 0, 4, 8 and 12 h, and observed the change in ITGB4 expression. Western blot assay showed that, with the extension of CHX treatment time, the expression of ITGB4 gradually decreased, while high NEDD4L expression accelerated the ITGB4 degradation (Fig. 4A-D). When cells were treated with protease inhibitor MG132, the ITGB4 degradation was partially blocked (Fig. 4E, F). These results were also verified in His-ub transfected ECA109 and TE13 cells. After MG132 treatment, the ITGB4 ubiquitination level increased, which was further enhanced through activating NEDD4L (Fig. 4G, H). These data suggest that NEDD4L mediates ITGB4 degradation via the ubiquitin-protease pathway. Cell cloning assay demonstrated that, TE13 and ECA109 cells with high NEDD4L expression had apparently reduced proliferation capacity compared with cells with low NEDD4L expression. After reversing ITGB4 expression, NEDD4L lost its ability to suppress cell proliferation (Fig. 4I). Transwell assay also confirmed that after restoring ITGB4 expression, NEDD4L lost its ability to suppress migration (Fig. 4J-L). The above results reveal that NEDD4L degrades ITGB4 via the ubiquitin-protease pathway to suppress the malignant phenotype of esophageal carcinoma.

### The HECT and Galx-β domains were required for NEDD4L-ITGB4 interaction

**Fig. 5:**
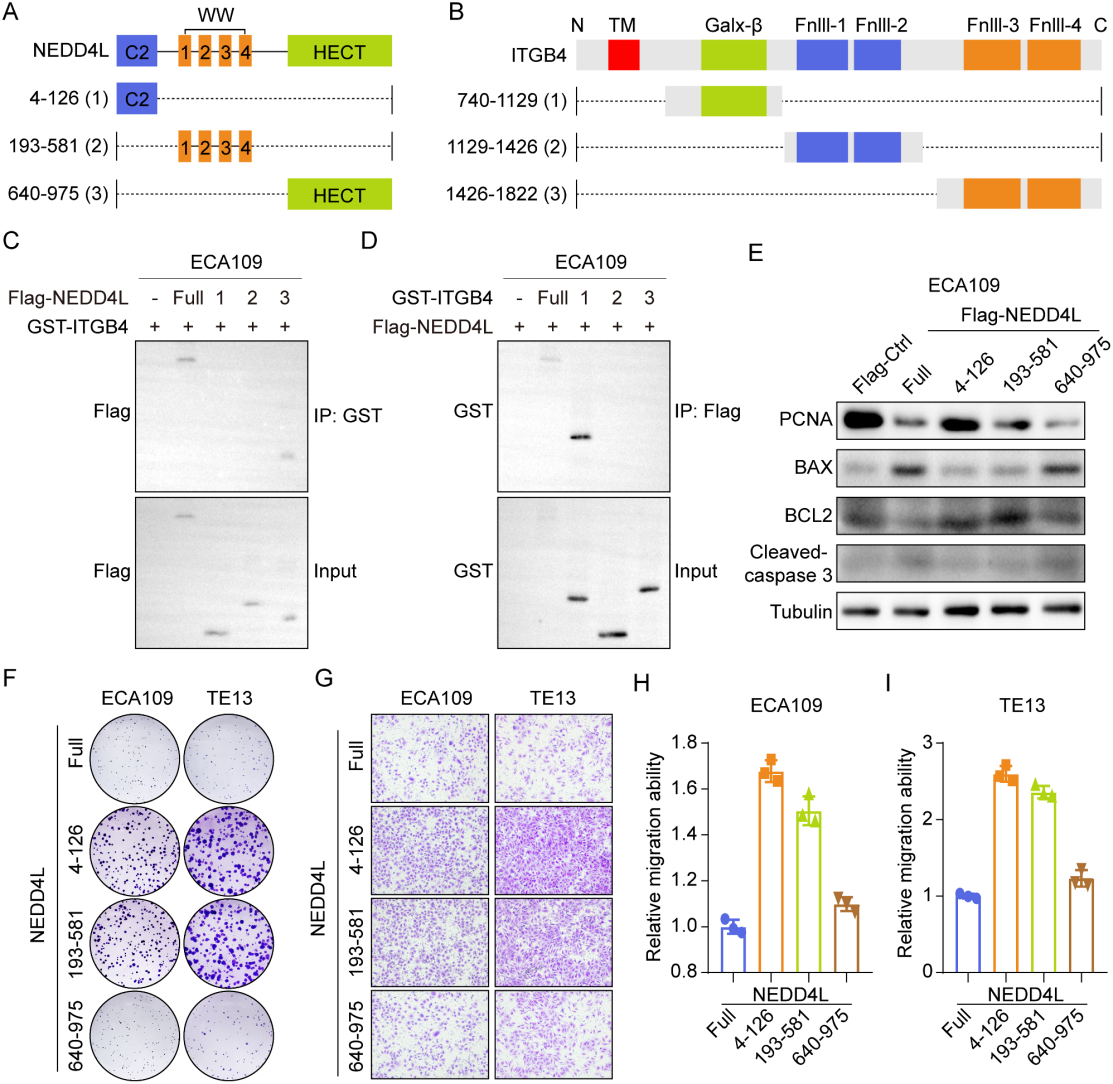
The HECT and Galx-β domains were required for NEDD4L-ITGB4 interaction. **(A)** Diagram of truncated forms of NEDD4L protein. Full: 1-975, C2: 4-126, WW: 193–581, HECT: 640–975. **(B)** Diagram of truncated forms of ITGB4 protein. Full: 1-1822, Galx-β: 740–1129, Fnll-1 and Fnll-2: 1129–1426, Fnll-3 and Fnll-4: 1426–1822. **(C)** ECA109 cell was co-transfected with GST-ITGB4 and different Flag-NEDD4L fragment plasmids (Full, C2, WW, or HECT). GST antibody was used to immunoprecipitate exogenous ITGB4 proteins, and NEDD4L fragment in the immunocomplexes was detected with Flag antibody via Western blot assay. **(D)** ECA109 cell was co-transfected with Flag-NEDD4L and different GST-ITGB4 fragment plasmids (Full, Galx-β, Fnll-1 and Fnll-2, or Fnll-3 and Fnll-4). Flag antibody was used to immunoprecipitate exogenous NEDD4L proteins, and ITGB4 fragment in the immunocomplexes was detected with GST antibody via Western blot assay. **(E)** Protein expression of PCNA, BAX, BCL2, Cleaved-Caspase3 were detected by western blot analysis after transfection with different Flag-NEDD4L fragment plasmids in ECA109 cell. **(F)** The representative images of Colony formation of TE13 and ECA109 cells in indicated groups. **(G-I)** The representative images and quantitative data of transwell assays in TE13 and ECA109 cells in indicated groups (*n* = 3)

To explore the key domain of NEDD4L that promoted the ITGB4 ubiquitination degradation, we synthesized different functional fragment plasmids of NEDD4L (Full: 1-975, C2: 4-126, WW: 193–581, HECT: 640–975) and ITGB4 (Full: 1-1822, Galx-β: 740–1129, Fnll-1 and Fnll-2: 1129–1426, Fnll-3 and Fnll-4: 1426–1822) (Fig. 5A, B). According to co-immunoprecipitation analysis results, the full-length ITGB4 only bound to the full-length NEDD4L and HECT domain (Fig. 5C). Meanwhile, the full-length NEDD4L only bound to the full-length ITGB4 and Galx-β domain (Fig. 5D). Such result suggests that the potential protein domains of NEDD4L-ITGB4 interaction are HECT and Galx-β domains. Western blot also demonstrated that the HECT domain in NEDD4L regulated the proliferation and apoptosis markers PCNA, BAX, BCL2, and Cleaved-caspase3 (Fig. 5E). From the perspective of function, cloning and Transwell assays also confirmed that the HECT domain of NEDD4L suppressed the proliferation and migration of esophageal carcinoma cell lines ECA109 and TE13 (Fig. 5F-I). These results indicate that the interaction between the HECT domain of NEDD4L and the Galx-β domain of ITGB4 promotes the ubiquitination degradation of ITGB4, thereby suppressing the malignant phenotype of esophageal carcinoma.

### The K915 site of ITGB4 was required for its ubiquitination by NEDD4L

**Fig. 6:**
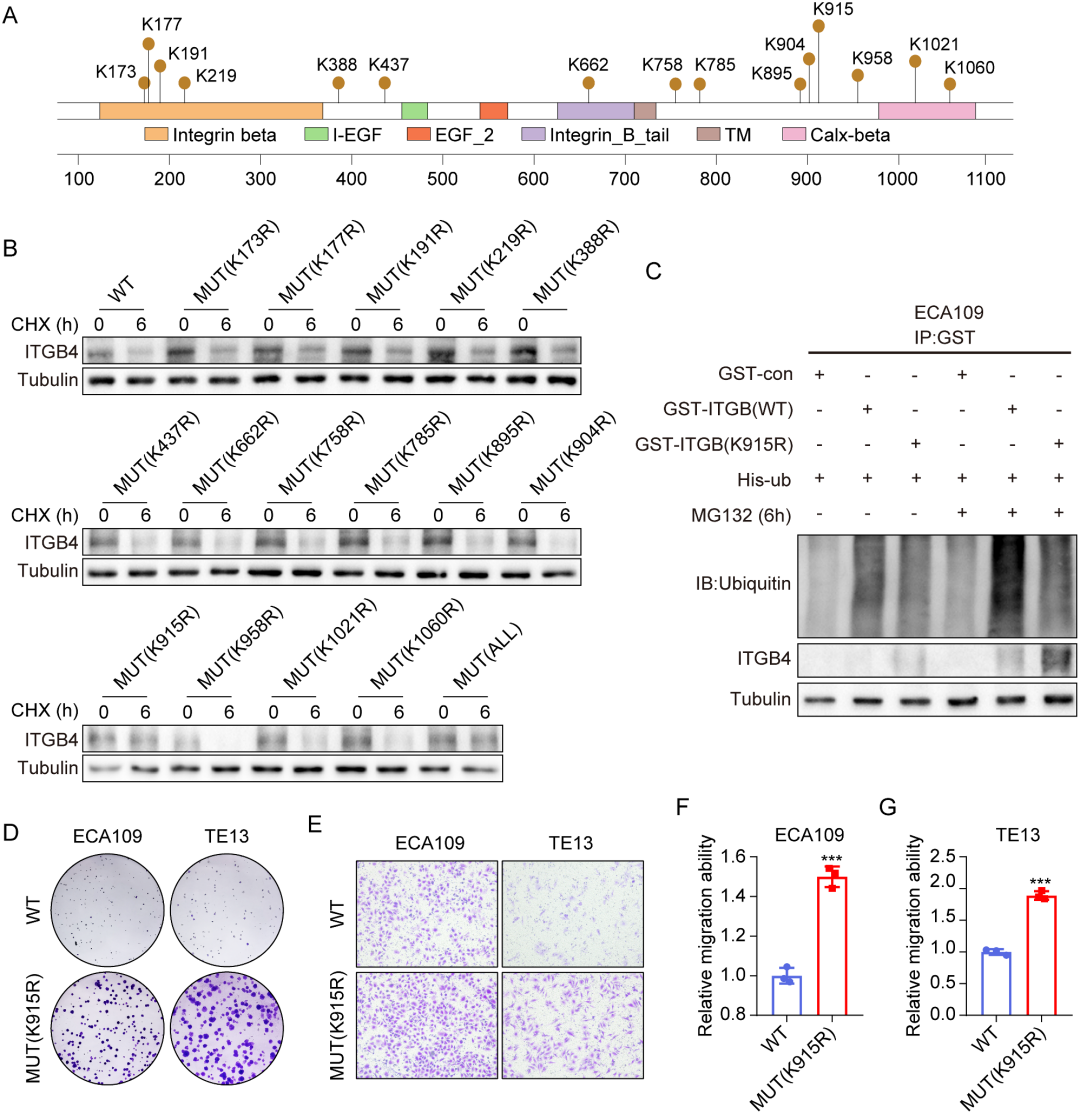
The K915 site of ITGB4 was required for its ubiquitination by NEDD4L. **(A)** Schematic representation of the potential ubiquitinated sites in ITGB4 protein. **(B)** The expressions of ITGB4 WT or mutants in ECA109 cells; by Western blot assay. **(C)** the ubiquitination and expression of Flag-ITGB4 (WT or K915R) in ECA109 cells; by Co-IP and western blot analysis. **(D)** The representative images of Colony formation of TE13 and ECA109 cells in indicated groups. **(E-G)** The representative images and quantitative data of transwell assays in TE13 and ECA109 cells in indicated groups (*n* = 3). (***P value < 0.001)

To identify the potential site needed for ITGB4 ubiquitination, we predicted 15 potential lysine sites of ITGB4 protein sequence (K173, K177, K191, K219, K388, K437, K662, K758, K785, K895, K904, K915, K958, K1021, K1060) (https://www.phosphosite.org/) (Fig. 6A). Subsequently, these 15 lysine sites were mutated separately or totally into arginine. Then, the ITGB4 wild type (WT) or mutant (MUT) plasmid was transfected into ECA109 cells, followed by CHX treatment for 0–6 h. Western blot assay suggested that cells transfected with K915R mutation or all point mutations were resistant to the CHX-accelerated ITGB4 degradation (Fig. 6B). To verify this, the WT or K915R mutation plasmid and ubiquitination plasmid His-ub were co-transfected into ECA109 cells and then cells were treated with CHX for 0–6 h, respectively. Western blot assay revealed that cells transfected with K915R mutation plasmid were resistant to NEDD4L-induced ITGB4 degradation (Fig. 6C). Cloning and Transwell assays also demonstrated that, compared with NEDD4L WT cells, NEDD4L (K915R) cells had enhanced proliferation and migration capacities (Fig. 6D-G). Our results suggest that the K915 site of ITGB4 is necessary for its ubiquitination degradation by NEDD4L.

### NEDD4L promoted ITGB4 ubiquitination degradation in vivo to suppress the growth and metastasis of esophageal carcinoma

**Fig. 7:**
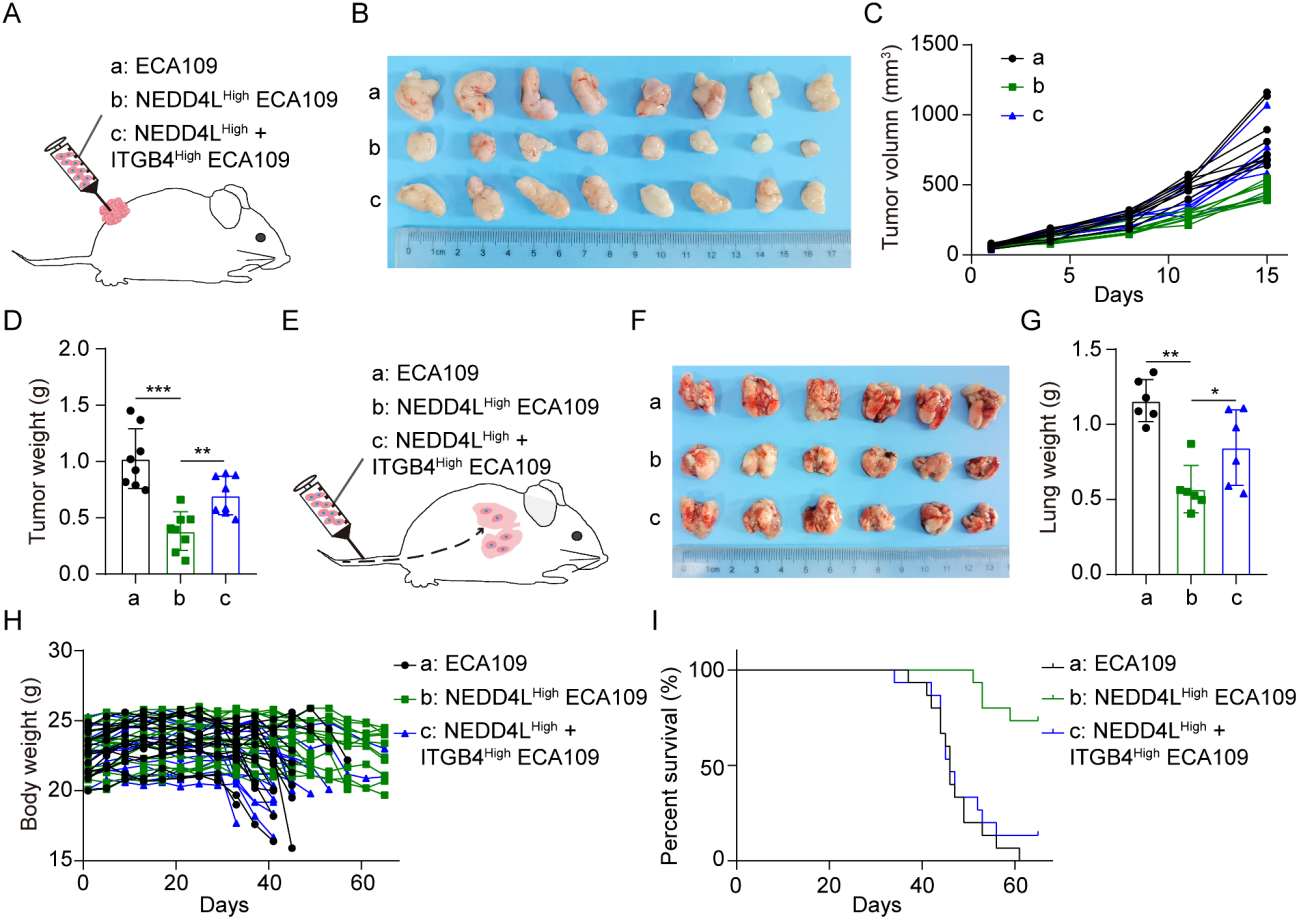
NEDD4L promoted ITGB4 ubiquitination degradation in vivo to suppress the growth and metastasis of esophageal carcinoma **(A-D) (A)** Schematic representation of the ECA109 cells ESCA-bearing model (*n* = 8 per group). The representative images of subcutaneous tumors **(B)**, tumor growth curves **(C)**, and tumor weight **(D)** in indicated groups. **(E-G) (E)** Schematic representation of the ECA109 cells ESCA lung metastasis model (*n* = 6). The representative lung metastasis images **(F)** and lung weight **(G)** in indicated groups. **(H-I)** ECA109 cells ESCA lung metastasis survival model. The body weights **(H)** and Kaplan-Meier survival curve **(I)** in indicated groups. (*n* = 15 per group). (*P value < 0.05; **P < 0.01; ***P < 0.001)

In order to validate the role of NEDD4L in promoting ITGB4 ubiquitination degradation in the growth and metastasis of esophageal carcinoma in vivo, we screened the high-NEDD4L expression ECA109 cells and the high-NEDD4L and high-ITGB4 expression ECA109 cells through the NEDD4L and ITGB4 high-expression lentivirus, and constructed the esophageal carcinoma tumor bearing and metastasis models. As revealed by the esophageal carcinoma tumor bearing model, high NEDD4L expression markedly suppressed tumor growth. While after restoring ITGB4 expression, NEDD4L lost its suppression on the growth of esophageal carcinoma (Fig. 7A-D). Similarly, the esophageal carcinoma metastasis model also suggested that, after restoring ITGB4 expression, NEDD4L lost its suppression on the metastasis of esophageal carcinoma (Fig. 7E-G). Moreover, we observed the effect of NEDD4L-mediated ubiquitination degradation of ITGB4 on the survival of mice by suppressing the metastasis of esophageal carcinoma. Our results suggested that, high NEDD4L expression markedly prolonged the survival of mice, while after reversing ITGB4 expression, NEDD4L lost its function in extending the survival of mice with esophageal carcinoma lung metastasis (Fig. 7H, I). The above results indicate that NEDD4L promotes ITGB4 ubiquitination degradation to effectively suppress the in vivo growth and metastasis of esophageal carcinoma.

## Discussion

Genome instability is one of the characteristics of tumor cells [24]. Under the endogenous or exogenous stress, the unstable expression of oncogene or tumor suppressor gene leads to the production of substantial heterogeneities inside the tumor, thus promoting tumor progression [25]. NEDD4L is regulated by multiple factors and molecules in the living body. CREB/CRTC2 [26] and miR-1 [27] can regulate NEDD4L expression through transcriptional regulation, while AUF1 [28] and 14-3-3 protein [29] regulate its expression via post-transcriptional regulation, thereby affecting biological functions. NEDD4L variation is discovered in nervous system tumors (like glioma) [30], respiratory system tumors (like lung cancer) [31], digestive system tumors (including gastric cancer, liver cancer, pancreatic cancer and intestinal cancer) [32–34], endocrine target organ tumors (breast cancer, ovarian cancer, cervical cancer and prostatic cancer) [35–38], and hematologic system tumors (leukemia and multiple myeloma) [39, 40], which has affected the progression of these tumors. We collected 117 esophageal carcinoma and para-carcinoma tissue samples from Jiangsu University Affiliated People’s Hospital and confirmed through Western blot and immunohistochemistry that, the expression of NEDD4L in ESCC tissue was markedly lower than that in para-carcinoma tissues. Besides, its expression further decreased with the increase in malignancy grade of esophageal carcinoma. Furthermore, as revealed by in vivo and in vitro growth and metastasis models, NEDD4L suppressed the growth and metastasis of esophageal carcinoma. Our findings in esophageal carcinoma are consistent with conclusions made in other tumor research, suggesting that the variation of NEDD4L in tumor affects the progression of esophageal carcinoma.

NEDD4L protein includes 3 domains, namely, the C2 domain at the N-terminal, the 4-WW domain in the middle, and the HECT domain at the C-terminal [41]. The major functions of C2 domain include binding to Ca2^+^ and protein-protein interaction. The WW domain plays a pivotal role in the recognition and binding of NEDD4L specific substrate. The HECT domain participates in catalyzing the polyubiquitin chain packaging, which involves the catalytic mechanism of the E2 ubiquitin binding site. Under normal condition, the mutual suppression of C2 domain and HECT domain can control the activity of each other. When Ca2^+^ in cells binds to the C2 domain, such balance is broken, thus activating the NEDD4L ubiquitin ligase activity [42]. According to our results in this study, NEDD4L exerts the role of E3 ubiquitin ligase activity to suppress the malignant phenotype of esophageal carcinoma.

NEDD4L is an ubiquitin ligase in the NEDD4 family, which has important functions in binding and regulating numerous membrane proteins [41]. In this study, co-immunoprecipitation assay and protein mass spectrometry qualitative analysis were conducted to screen NEDD4L specific binding proteins. Subsequently, based on the bioinformatics platform UbiBrowser, the esophageal carcinoma TCGA database and GEO database (GSE53625), we screened the ITGB4 protein, which was related to the progression of esophageal carcinoma, and could be degraded by NEDD4L-mediated ubiquitination. ITGB4 (integrin β4 receptor) is the transmembrane receptor that mediates the connection between cells and their external environment, which participates in regulating cellular communication, cell cycle, cell morphology and cell movement, and exerts an indispensable role in the occurrence and development of numerous diseases, especially malignant tumors [43]. The expression of ITGB4 is up-regulated in multiple malignant tumors such as breast cancer [44], lung cancer [45] and liver cancer [46], which is related to the dismal patient prognosis. For instance, ITGB4 promotes triple-negative breast cancer to resist DNA damage via TNFAIP2/IQGAP1/RAC1, and thus accelerates the drug resistance of tumor cells [44]. ITGB4 regulates the expression of transcription factor Slug to affect the epithelial-mesenchymal transition (EMT) of liver cancer cells, and promote tumor cell invasion [47]. Interestingly, it was also confirmed in this study that the protein expression of TNFAIP2/IQGAP1/RAC1 and Slug was significantly decreased after NEDD4L^high^ treatment. While after restoring ITGB4 expression, NEDD4L lost its suppression on the expression of TNFAIP2/IQGAP1/RAC1 and Slug (Fig. S3). These results indicate that NEDD4L may regulate TNFAIP2/IQGAP1/RAC1 and Slug by promoting ITGB4 ubiquitination degradation. ITGB4 possesses a TM domain, a Galx-β domain, 2 pairs of type-III FN repeated fragments, and the CS fragment that connects FN fragments. The 2 pairs of type-III FN fragments interact with plectin, which has the highest abundance in the cytoplasmic solute, and is later connected onto the keratin cytoskeleton, thus endowing ITGB4 with the stable adhering capacity. Galx-β domain and FN fragments can bind to multiple keratin cytoskeletons to affect the transmission of intracellular signal mediators, thus affecting cell function. In this study, ITGB4 was related to the poor prognosis of esophageal carcinoma. The HECT domain of NEDD4L specifically bound to the Galx-β domain of ITGB4 to maintain the stable interaction between these two proteins. Subsequently, NEDD4L modified the K915 site of ITGB4 through ubiquitination to promote the ubiquitination degradation of ITGB4.

**Fig. 8:**
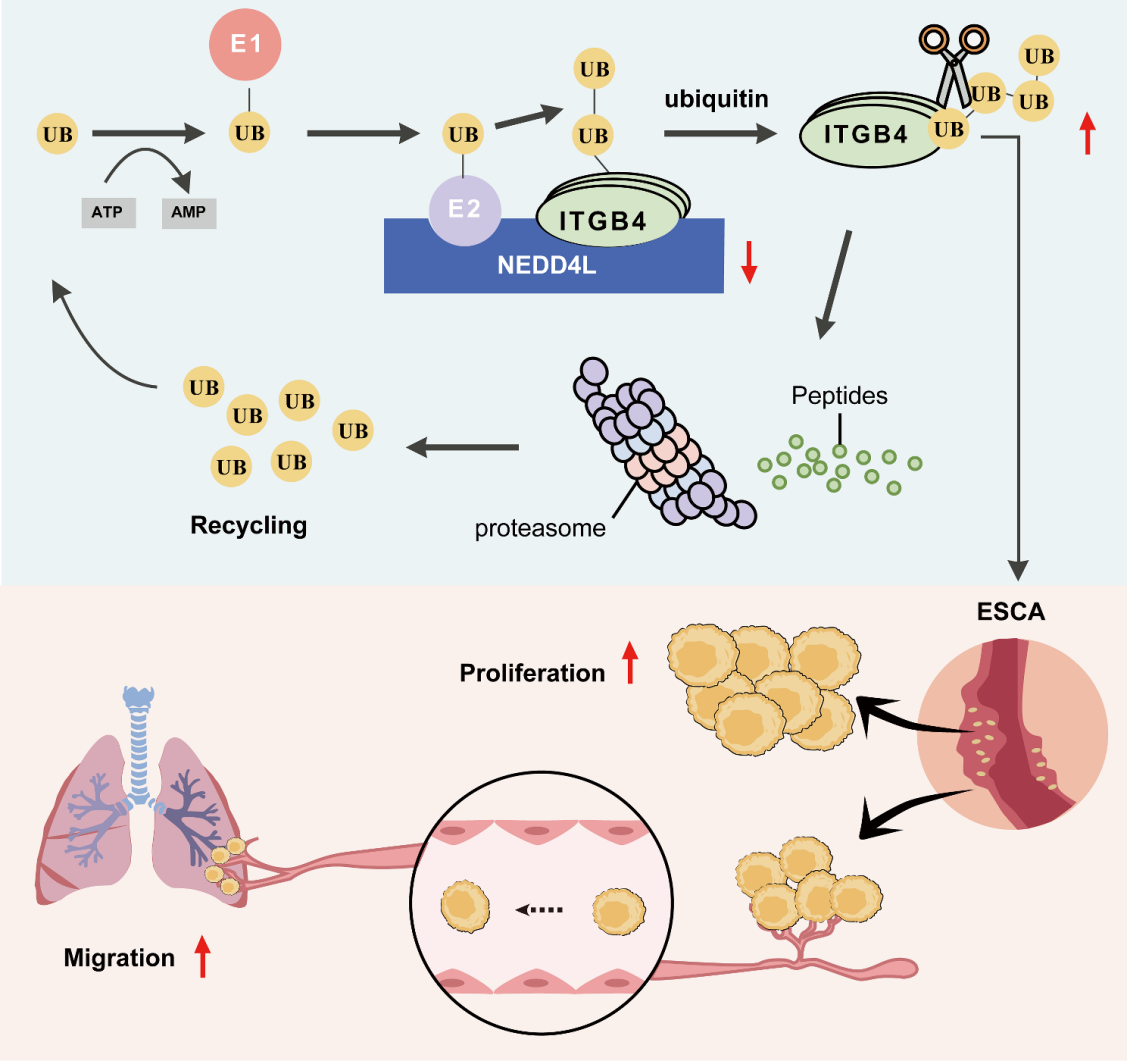
The mechanism of NEDD4L in inhibiting esophageal carcinoma progression

To sum up, this study discovers from esophageal carcinoma TCGA data and GEO data that, the expression of NEDD4L in esophageal carcinoma is apparently lower than that in atypical hyperplastic esophageal tissue and esophageal squamous epithelium. Moreover, patients with high expression of NEDD4L in esophageal carcinoma tissue have longer progression-free survival than those with low expression. In experiments in vivo and in vitro, NEDD4L suppresses the proliferation and migration of esophageal carcinoma cells. Mechanically, the HECT domain of NEDD4L specifically binds to the Galx-β domain of ITGB4, which modifies the K915 site of ITGB4 through ubiquitination, and promotes the ubiquitination degradation of ITGB4, thus suppressing the malignant phenotype of esophageal carcinoma (Fig. 8). This study suggests that NEDD4L may be the early diagnostic molecular biomarker and potential therapeutic target for esophageal carcinoma.

## Materials and methods

### ESCC patient samples

In total, 117 surgical tissue samples of esophageal carcinoma patients were collected from Jiangsu University Affiliated People’s Hospital (Zhenjiang, Jiangsu). All tissues were pathologically confirmed after surgery. The patients did not receive other treatments like chemotherapy or radiotherapy before surgery. The collection of all tissue samples was completed within 5 min after tumor excision, and preserved in the liquid nitrogen for a long time. The present study was approved by the Ethics Committee of Jiangsu University Affiliated People’s Hospital (approval No.: K-20,220,098-Y). All patients signed the informed consents.

### Cell culture

Human esophageal carcinoma cells TE13 and ECA109 were purchased from Type Culture Collection of the Chinese Academy of Sciences (Shanghai, China). All cells were cultured in RPMI1640 (GIBCO, USA) medium supplemented with 1% streptomycin and penicillin and 10% fetal bovine serum, and incubated in the 37 ℃ and 5% CO_2_ incubator.

### Plasmid transfection

The pcDNA3.1-Flag-NEDD4L full-length and fragment plasmids (Full:1-975, C2: 4-126, WW: 193–581, HECT: 640–975), and pcDNA3.1-GST-ITGB4 full-length and fragment plasmids (Full:1-1822, Galx-β: 740–1129, Fnll-1 and Fnll-2: 1129–1426, Fnll-3 and Fnll-4: 1426–1822) were constructed by YouBao Biology (Changsha, China). NEDD4L and ITGB4 overexpression lentivirus, together with NEDD4L point mutation plasmids (K173, K177, K191, K219, K388, K437, K662, K758, K785, K895, K904, K915, K958, K1021, K1060) were constructed by Corues Biotechnology (Nanjing, China). In line with manufacturer’s instructions, the Lipofectamine 3000 (Invitrogen) was used to transfect the plasmids into cells.

### Western blot assay and co-immunoprecipitation assay

Cell samples were lysed with RIPA lysate (P0013C, Beyotime, Shanghai, China) for 30 min to extract the total cellular protein. For tissue samples, they were grinded into pieces with liquid nitrogen, and then lysed with the enhanced RIPA lysate (P0013B, Beyotime, Shanghai, China) for 2 h to extract the total tissue protein. The total protein concentration was quantified using the BCA protein concentration detection kit (P0012, Beyotime, Shanghai, China). Thereafter, proteins were separated through SDS-polyacrylamide gel electrophoresis (SDS-PAGE) and transferred onto polyvinylidene fluoride (PVDF) membranes (Millipore, Darmstadt, Germany). Afterwards, the membranes were blocked with 5% defatted milk powder for 1 h. Then, the membranes were incubated with primary antibodies overnight at 4 ℃, washed with PBST for 10 min 5 times, and further subject to incubation with horse radish peroxidase (HRP)-conjugated secondary antibodies for 1 h. Subsequently, signals were observed using the enhanced chemiluminescence (ECL) detection kit (Vazyme, Nanjing, China). The antibodies used in the experiments were shown below, NEDD4L (13690-1-AP, proteintech, Wuhan, China); ITGB4 (21738-1-AP, proteintech, Wuhan, China); TNFAIP2 (25649-1-AP, proteintech, Wuhan, China); IQGAP1 (22167-1-AP, proteintech, Wuhan, China); RAC1 (24072-1-AP, proteintech, Wuhan, China); Slug (12129-1-AP, proteintech, Wuhan, China); PCNA (13,110 S, CST, Massachusetts, USA); BAX(2772 S, CST, Massachusetts, USA); BCL2 (3498 S, CST, Massachusetts, USA); cleaved caspase3 (9664 S, CST, Massachusetts, USA); Tubulin (AF2827, Beyotime, Shanghai, China), horse radish peroxidase (HRP)-conjugated secondary antibodies (A0208/A0216, Beyotime, Shanghai, China). The assay was repeated thrice.

As for co-immunoprecipitation assay, the treated cells were collected, lysed with the TNE buffer that contained the protease inhibitor (50 mM Tris-HCl, 150 mM NaCl, 1% NP40, pH7.4), and incubated with specific primary antibodies overnight at 4 °C. The protein A/G agarose beads (sc-2003, Santa Cruz, California, USA) were supplemented to incubate the cells for another 2 h at 4 °C. Then, the agarose bead-antibody-protein complex was collected, washed with TNE buffer 5 times, and added with 2×SDS loading buffer to detect protein levels through Western blot. In the ubiquitination assay, cells were treated with MG132 (10 µM) for 6 h before collection, and detected through Western blot assay.

### Immunohistochemistry

The mouse tumor tissue samples were fixed in formalin solution for 24 h, embedded in paraffin, and sliced into sections (paraffin slicer, HM340E, Thermo Fisher Scientific). Human esophageal carcinoma tissue samples were prepared into tissue chips by Bioaitech Co.,Ltd (Xi’an, China). After oven drying and antigen retrieval, the sections were blocked with 10% normal goat serum at room temperature for 30 min, and incubated with specific primary antibodies at 4 °C overnight. After DAB staining, the images were scanned using the pathological section scanner (Pannoramic MIDI). The antibodies utilized in the experiment included NEDD4L (13690-1-AP, proteintech, Wuhan, China); ITGB4 (21738-1-AP, proteintech, Wuhan, China); cleaved caspase3 (9664 S, CST, Massachusetts, USA); and ki67 (GB111141-100, Servicebio, Wuhan, China).

### Cell proliferation assay

ECA109 and TE13 cells were treated according to experimental design. In brief, 1000 cells were inoculated into the 6-well plate, and cultured in the cell incubator, with medium change every 3 days. According to cell growth condition, ECA109 and TE13 cells were collected at 10 and 14 days, separately, and fixed with methanol for 1 h. Later, cells were stained with crystal violet dye (C0121, Beyotime, Shanghai, China) for 1 h, photographed and counted.

### Transwell assay

Cell migration ability was determined in the Transwell chambers (pore size, 8 μm; 3472, Corning, New York, USA). At one day prior to the experiment, a layer of FN fibronectin (F8180, Solarbio, Beijing, China) was coated onto the bottom of Transwell chamber, and the chamber was then placed into the cell incubator. On the day of experiment, 30,000 treated ECA109 and TE13 cells were inoculated into the serum-free medium in the upper chamber, while medium including 10% FBS was added into the lower chamber. ECA109 and TE13 cells were collected after cultured 24 h and 48 h, separately, and fixed with methanol for 1 h. Subsequently, cells were stained with crystal violet dye (C0121, Beyotime, Shanghai, China) for 1 h, photographed and counted.

### Flow cytometry

To detect cell apoptosis, the Annexin V-FITC cell apoptosis detection kit (C1062M, Beyotime, Shanghai, China) was used for cell staining in line with manufacturer’s instructions. In brief, cells were treated according to the experimental design, then collected (100,000) with the EDTA-free trypsin, and stained with Annexin V-FITC. Thereafter, cell apoptosis rate was analyzed with the flow cytometer (Beckman Coulter, Inc. California, USA).

### Animal experiments

The 5-week-old male BALB/c nude mice were purchased from Gempharmatech Co., Ltd (Nanjing, China), and raised at the Laboratory Animal Center of Nantong University) (SPF facilities). All animal experiments in this study were performed strictly following the protocols approved by the Animal Care and Use Committee of Nantong University (approval No.: S20220224-006).

To construct the tumor-bearing model, the 5-week-old male BALB/c nude mice were grouped according to their body weights at one day prior to the experiment, with 8 mice in each group. On the date of experiment, ECA109 cells at the logarithmic growth phase were collected and injected (5*10^6^/100 µl) subcutaneously in the right side of mouse back. When the average tumor volume reached about 50 mm^3^, the mouse body weight and tumor volume were recorded every three days. Mice were euthanatized with the average tumor volume reaching about 1000 mm^3^ in any one group, and the model was terminated. The tumor tissue was collected, weighed, recorded, and preserved in the − 80 °C refrigerator for subsequent assay.

To build the lung metastasis model through tail vein injection, the 5-week-old male BALB/c nude mice were grouped according to their body weights at one day prior to the experiment, with 6 mice in each group. On the date of experiment, ECA109 cells at the logarithmic growth phase were collected and injected (2*10^6^/100 µl) into each mouse through the tail vein. Mice were euthanatized at 6 weeks later, lung tissue was dissected and weighed, and lung metastasis was observed. Subsequently, lung tissue was preserved at the − 80 °C refrigerator for subsequent assay. For the lung metastasis survival model through tail vein injection, the death of mice was recorded every day after they were injected with ECA109 cells via the tail vein.

### Protein docking analysis

The three-dimensional protein structures of NEDD4L and ITGB4 were predicted based on AlphaFold Protein Structure Database (https://alphafold.ebi.ac.uk/). Subsequently, the docking analysis of NEDD4L and ITGB4 protein was simulated through the HDOCK server (http://hdock.phys.hust.edu.cn/). The confidence score of the two-protein binding model was calculated based on the iterative scoring function ITScorePP or ITScorePR, Confidence_score = 1.0/[1.0 + e0.02*(Docking_Score + 150)]. When the confidence score is greater than 0.7, it is considered that there is an interaction between the two proteins. As shown in Fig. 3B, the confidence score of the interaction model between NEDD4L and ITGB4 proteins is 0.9211, indicating that there is an interaction between the two proteins.

### Public database and bioinformatics analysis

The esophageal carcinoma and para-carcinoma expression datasets GSE46452 and GSE26886 were downloaded from GEO database (https://www.ncbi.nlm.nih.gov/geo/). According to UALCAN (https://ualcan.path.uab.edu/index.html), the effect of high or low expression of NEDD4L on the survival of esophageal carcinoma patients was analyzed based on the TCGA-esophageal carcinoma data. The correlation of NEDD4L and ITGB4 expression was determined by R2: Genomics Analysis and Visualization Platform (http://r2.amc.nl).

### Statistical analysis

GraphPad Prism 8 and SPSS 21.0 (IBM, SPSS, Chicago, IL, USA) software were utilized for data analysis. Experimental data were expressed as means ± standard deviation. The Student’s t-test was applied in analyzing differences between two groups, whereas one-way analysis of variance was adopted to analyze differences among multiple groups. *P* < 0.05 stood for significant difference (*P-value < 0.05; **P < 0.01; ***P < 0.001; ns, no significance).

### Electronic supplementary material

Below is the link to the electronic supplementary material.

Supplementary Material 1

Supplementary Material 2

## Data Availability

All data in the manuscript and supplementary material are available.
