# Supplementary material for: NEDD4L mediates ITGB4 ubiquitination and degradation to suppress esophageal carcinoma progression
Source: Cell Commun Signal. 2024 Jun 3;22:302. doi: 10.1186/s12964-024-01685-9 (PMC11145805; doi:10.1186/s12964-024-01685-9)

**NEDD4L mediates ITGB4 ubiquitination and degradation to suppress esophageal carcinoma progression**

Yijun Shi^1 §^, Na Fang^2 §^, Yutong Wu^3 §^, Huiwen Xu^3^, Anhui Ning^3^, Wendi Zhang^3^, Yiran Liu^3^, Xiaobo Tao^3^, Qiong Chen^3^, Tian Tian^3^, Lei Zhang^3^, Minjie Chu^3^*, Jiahua Cui^3 4^*

**Supplementary Materials**

Supplementary Table 1. Correlation between NEDD4L expression and clinicopathological features of ESCC.

Supplementary Table 2. Correlation between ITGB4 expression and clinicopathological features of ESCC.

Supplementary Figure 1. Screening for Ubiquitinated Proteins of NEDD4L.

Supplementary Figure 2. The predicted protein structure of NEDD4L and ITGB4.

Supplementary Figure 3. The protein expression of TNFAIP2/IQGAP1/RAC1 and Slug signaling.

Supplementary Table 1. Correlation between NEDD4L expression and clinicopathological features of ESCC (n = 96).

| parameter | Number of patients | Low expression of NEDD4L  (%) | High expression of NEDD4L  (%) | *P* value |
| --- | --- | --- | --- | --- |
| Age (year) |  |  |  | 0.133 |
| ≤60 | 33 | 13 (39.39%) | 20 (60.61%) |  |
| ＞60 | 63 | 35 (55.56%) | 28 (44.44%) |  |
| Gender |  |  |  | 0.178 |
| Female | 28 | 17 (60.71%) | 11 (39.29%) |  |
| Male | 68 | 31 (45.59%) | 37 (54.41%) |  |
| Grade |  |  |  |  |
| I | 18 | 7 (38.89%) | 11 (61.11%) | 0.036* |
| II | 50 | 21(42.00%) | 29(58.00%) |  |
| III | 22 | 16 (72.73%) | 6 (27.27%) |  |
| Stage |  |  |  | 0.528 |
| I | 5 | 2 (40.00%) | 3(60.00%) |  |
| II | 44 | 22 (50.00%) | 22 (50.00%) |  |
| III | 45 | 22 (48.89%) | 23 (51.11%) |  |
| IV | 2 | 2 (100%) | 0 |  |
| Lymphatic metastases |  |  |  | 0.683 |
| Yes | 46 | 24 (52.17%) | 22 (47.83%) |  |
| No | 50 | 24 (48.00%) | 26 (52.00%) |  |

Supplementary Table 2. Correlation between ITGB4 expression and clinicopathological features of ESCC (n = 96).

| parameter | Number of patients | Low expression of ITGB4  (%) | High expression of ITGB4  (%) | *P* value |
| --- | --- | --- | --- | --- |
| Age (year) |  |  |  | 0.671 |
| ≤60 | 33 | 17 (51.52%) | 16 (48.48%) |  |
| ＞60 | 63 | 28 (44.44%) | 32 (50.79%) |  |
| Gender |  |  |  |  |
| Female | 28 | 14 (50.00%) | 14 (50.00%) | 1.000 |
| Male | 68 | 34 (50.00%) | 34 (50.00%) |  |
| Grade |  |  |  |  |
| I | 18 | 11 (61.11%) | 7 (38.89%) | 0.021* |
| II | 50 | 26(52.00%) | 24(48.00%) |  |
| III | 22 | 7 (31.82%) | 15 (68.18%) |  |
| Stage |  |  |  | < 0.001* |
| I | 5 | 4 (80.00%) | 1(20.00%) |  |
| II | 44 | 22 (50.00%) | 22 (50.00%) |  |
| III | 45 | 22 (48.89%) | 23 (51.11%) |  |
| IV | 2 | 0 | 2(100%) |  |
| Lymphatic metastases |  |  |  | 0.838 |
| Yes | 46 | 22 (47.83%) | 24 (52.17%) |  |
| No | 50 | 26 (52.00%) | 24 (48.00%) |  |

Supplementary Figure 1. Screening for Ubiquitinated Proteins of NEDD4L.


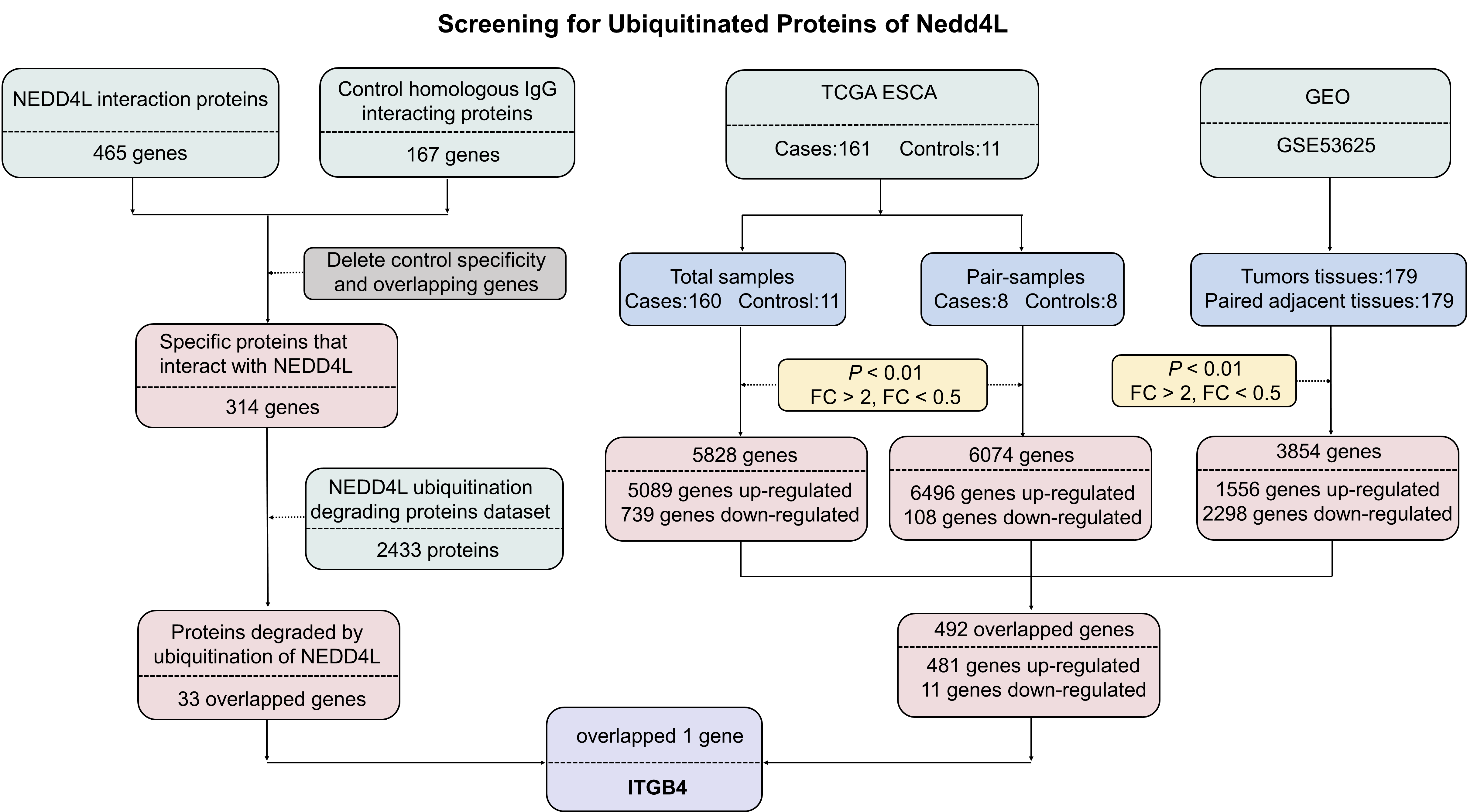


Supplementary Figure 2. The predicted protein structure of NEDD4L and ITGB4.


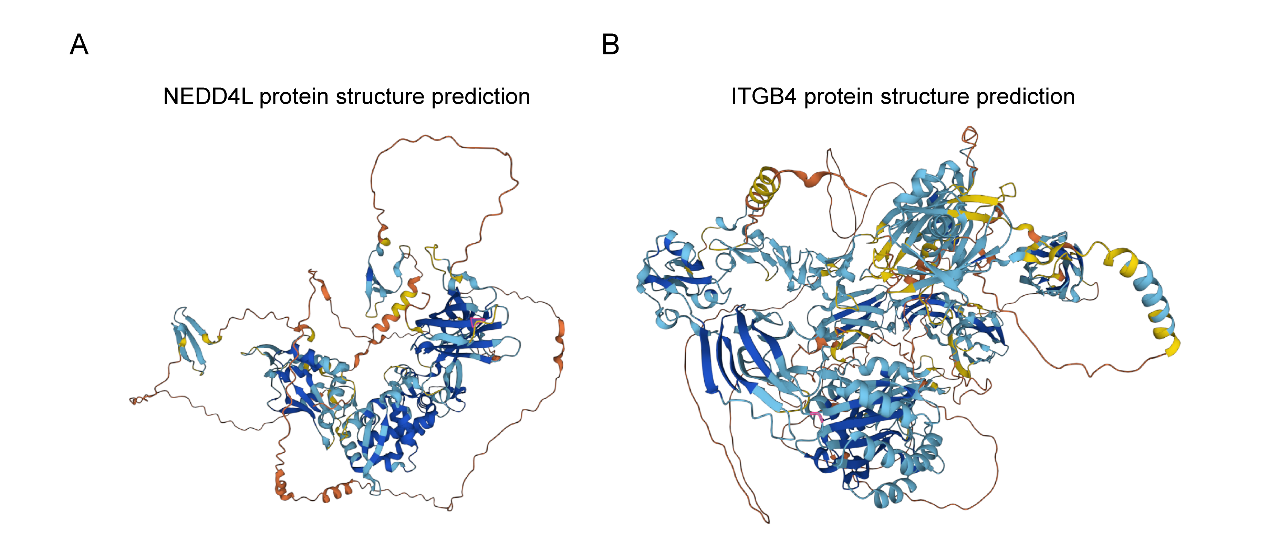


Supplementary Figure 3. The protein expression of TNFAIP2/IQGAP1/RAC1 and Slug signaling.


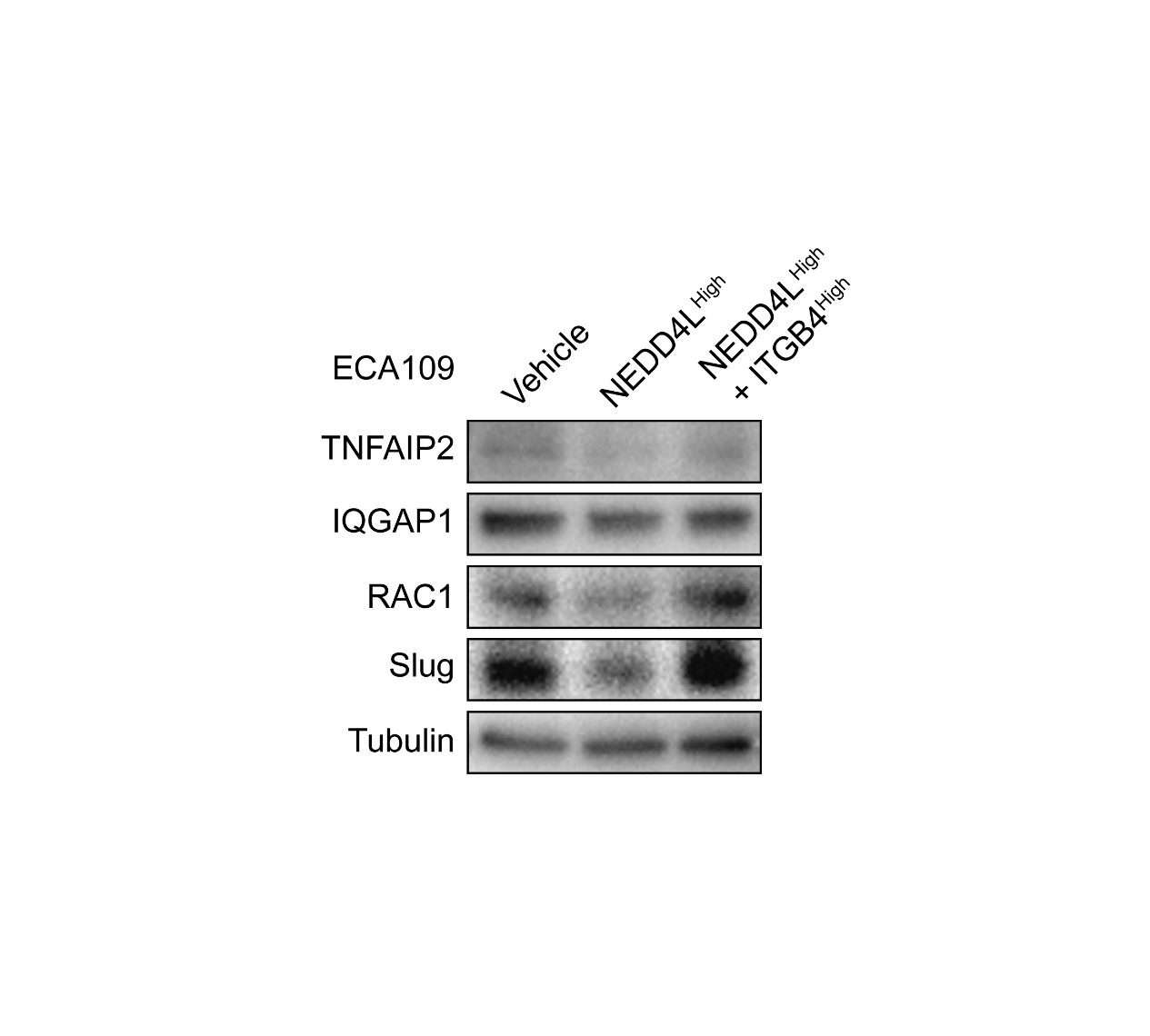

Supplement: Supplementary file 1 — Supplementary Material 1 [file 12964_2024_1685_MOESM1_ESM.docx]
