# Supplementary material for: NEDD4L mediates ITGB4 ubiquitination and degradation to suppress esophageal carcinoma progression
Source: Cell Commun Signal. 2024 Jun 3;22:302. doi: 10.1186/s12964-024-01685-9 (PMC11145805; doi:10.1186/s12964-024-01685-9)

Figure 1E-the full length uncropped original western blots

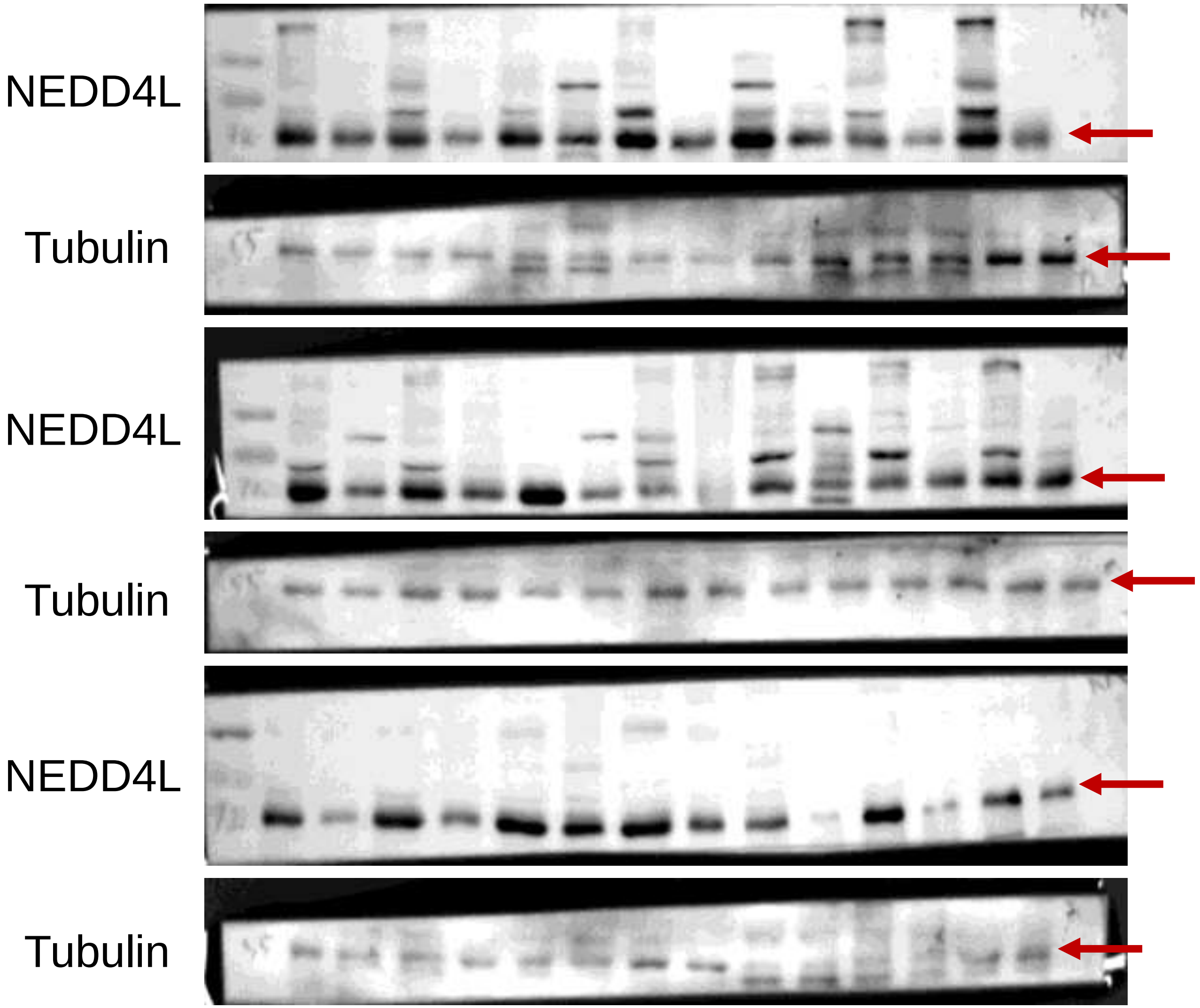

Figure 2-the full length uncropped original western blots

Figure 2A

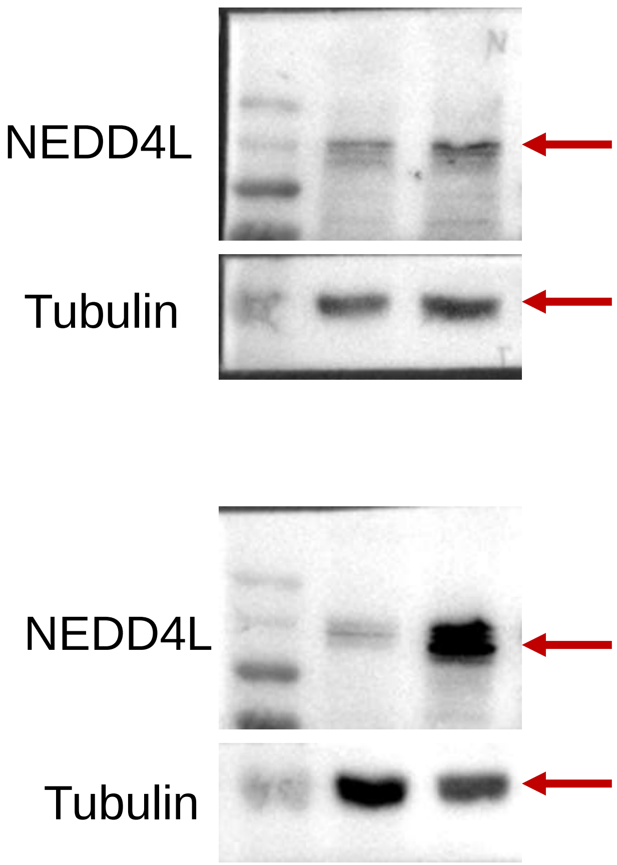

Figure 2I

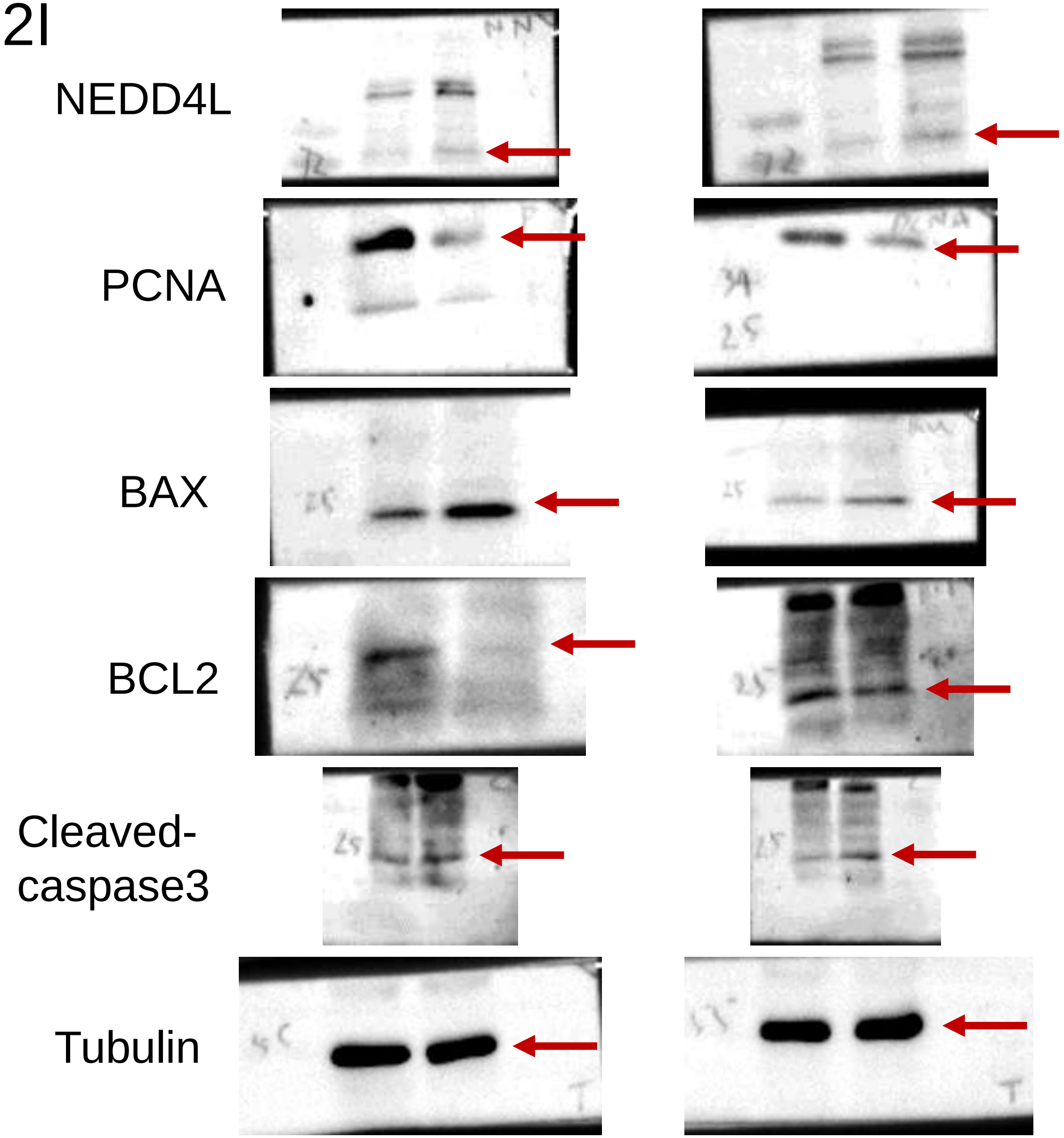

Figure 3C-the full length uncropped original western blots

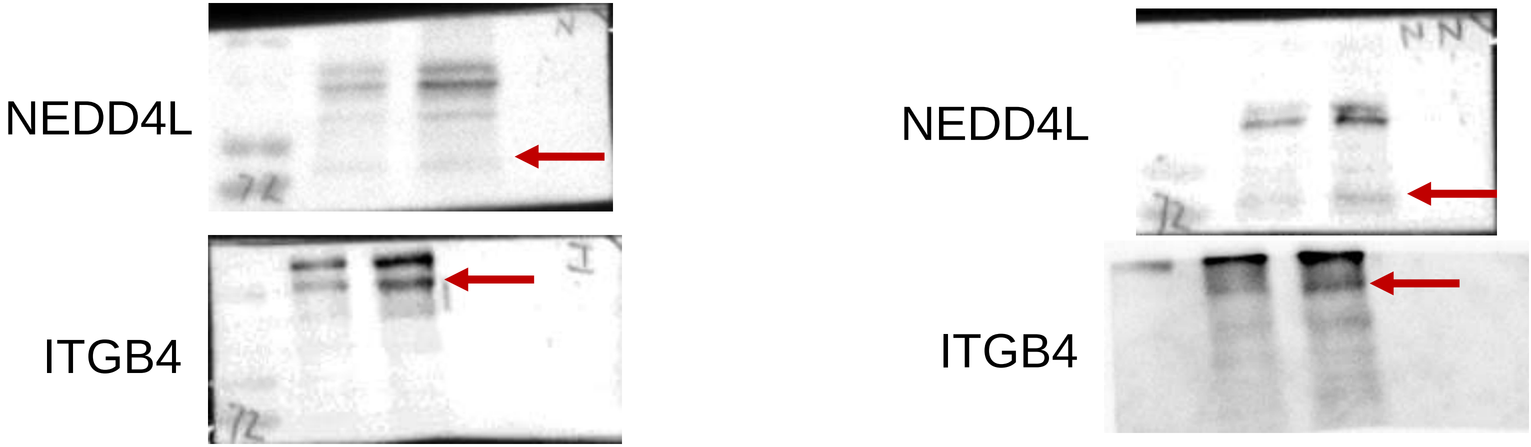

Figure 4-the full length uncropped original western blots

Figure 4A

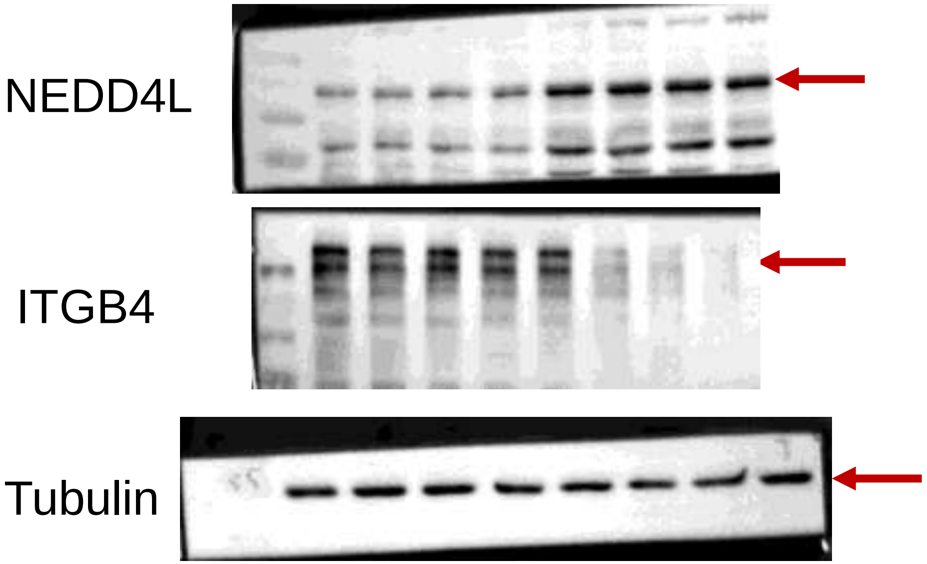

Figure 4C

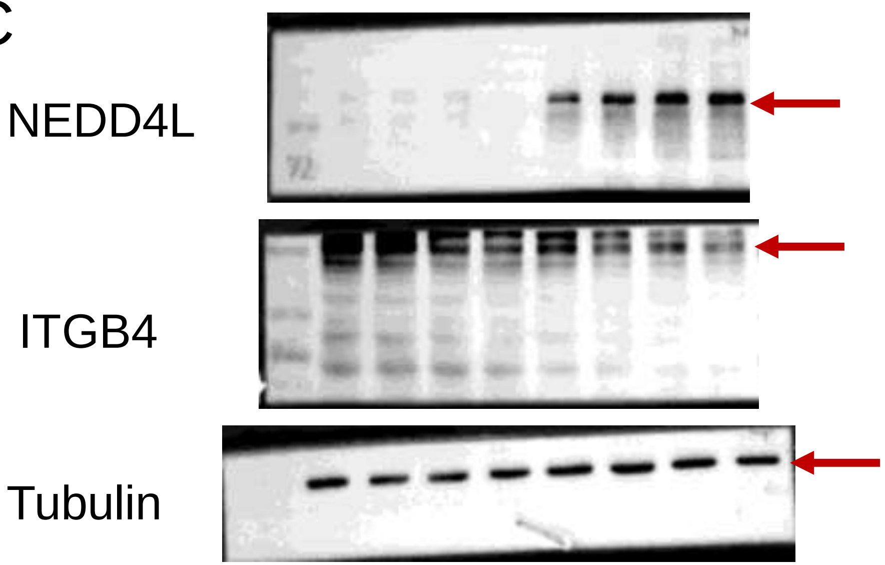

Figure 4E

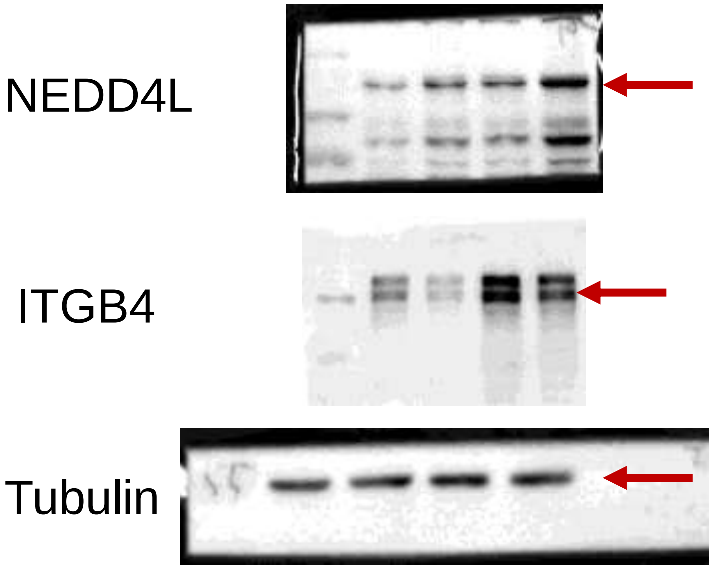

Figure 4F

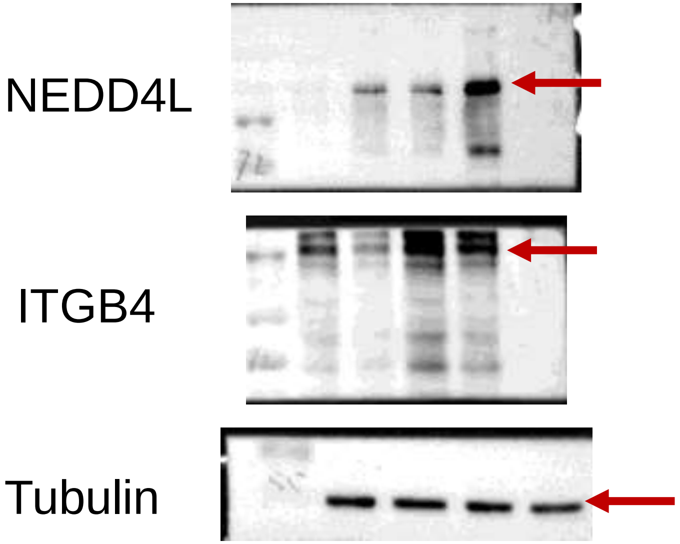

Figure 4-the full length uncropped original western blots

Figure 4G

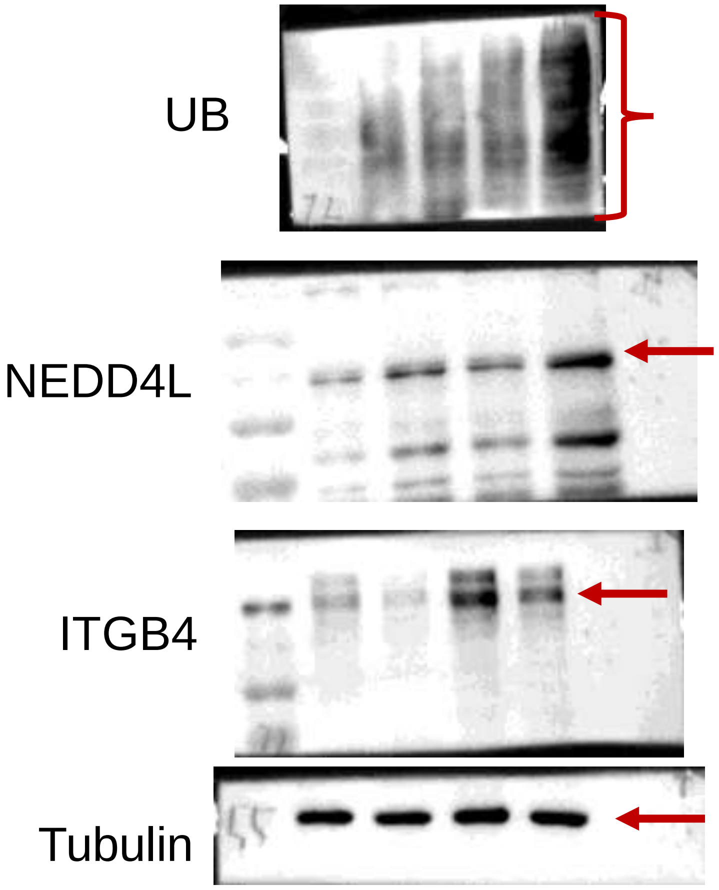

Figure 4H

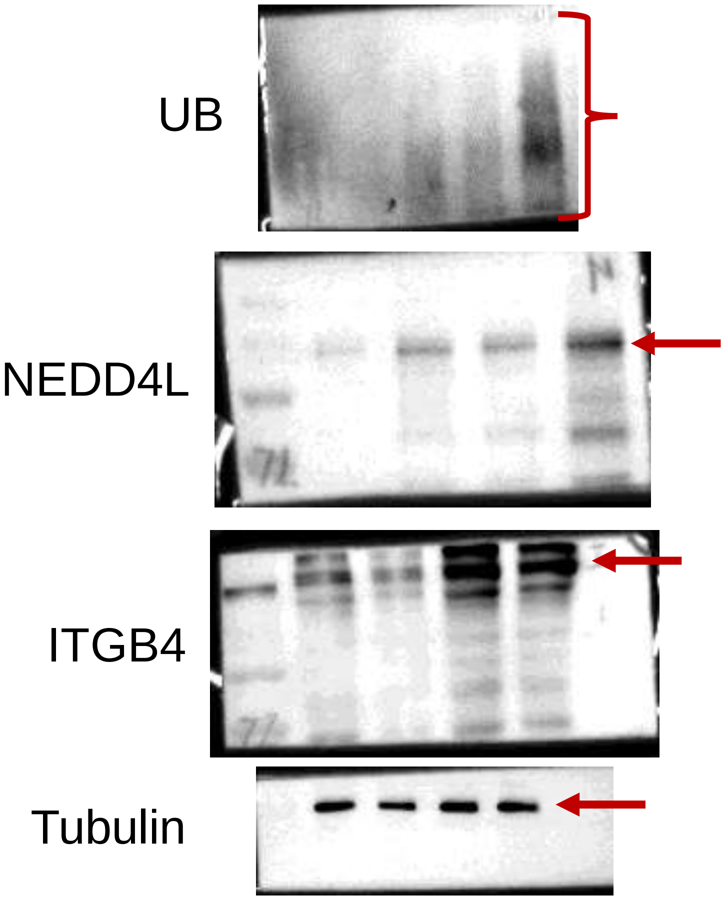

Figure 5-the full length uncropped original western blots

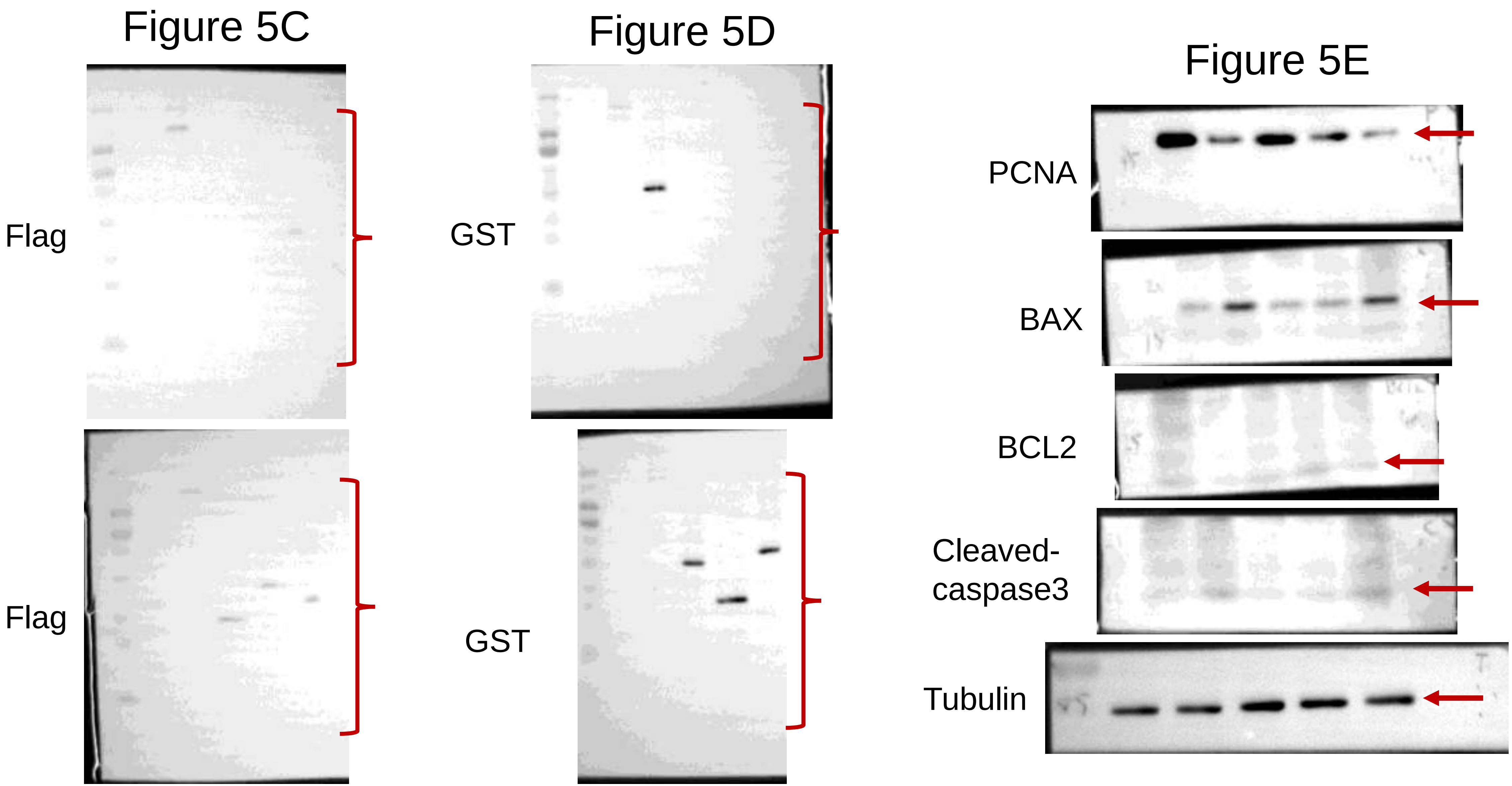

Figure 6-the full length uncropped original western blots

Figure 6B

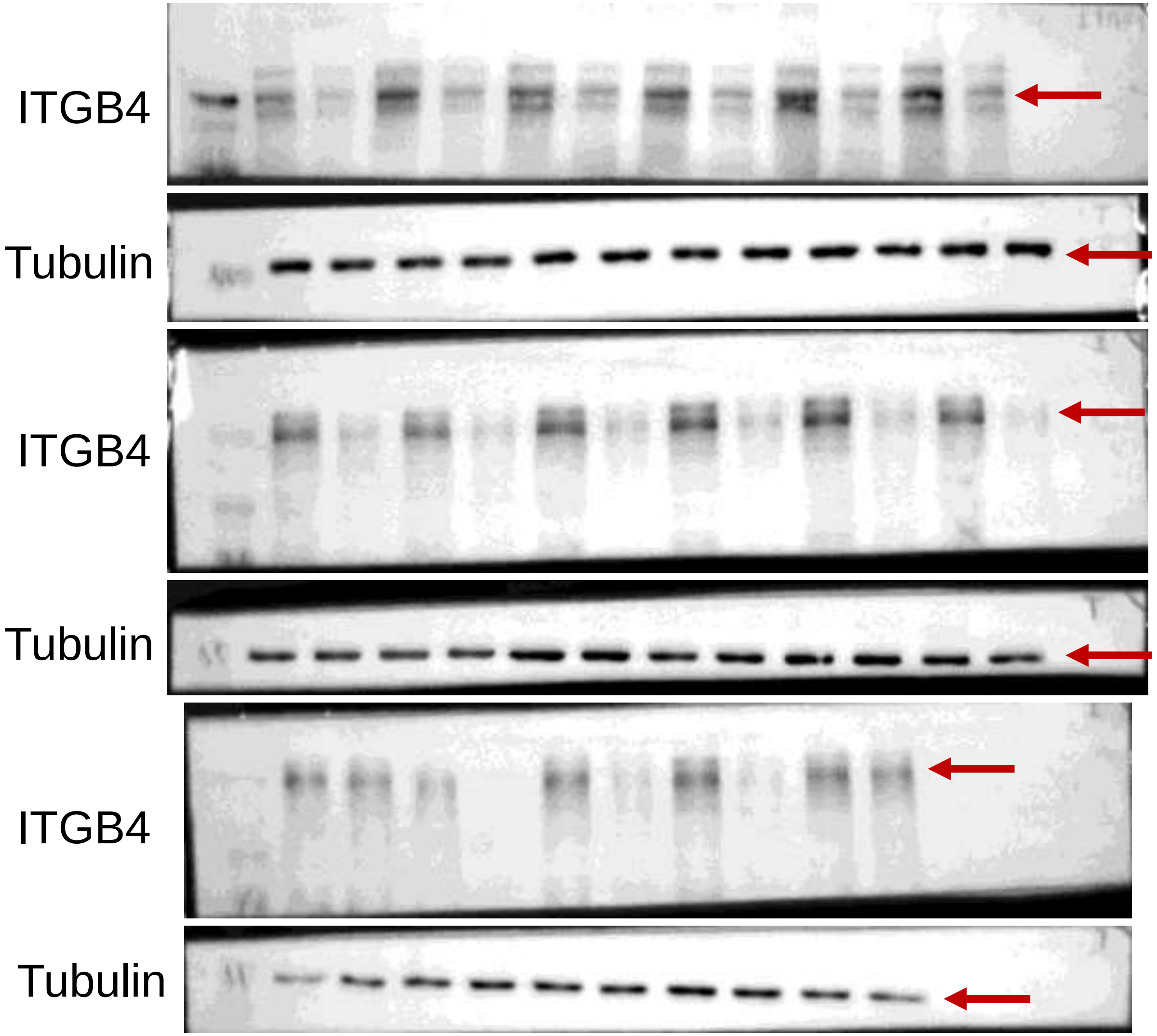

Figure 6C

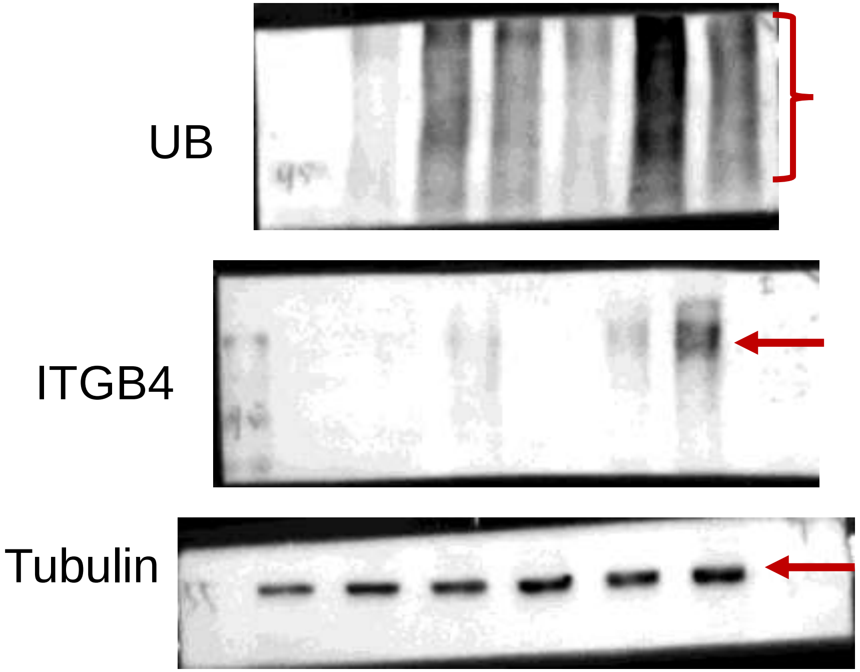

Supplementary Figure 3-the full length uncropped original western blots

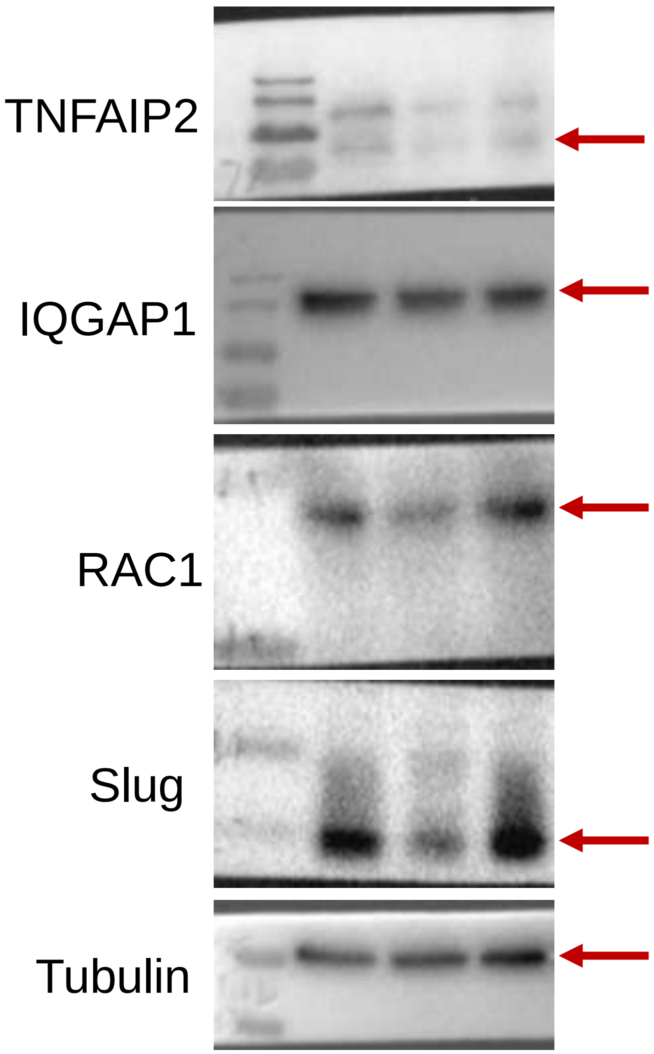

Supplement: Supplementary file 2 — Supplementary Material 2 [file 12964_2024_1685_MOESM2_ESM.pdf]
